# Distinct lipid and hormonal signatures reveal divergent responses to *Erysiphe necator* in resistant and susceptible grapevine genotypes

**DOI:** 10.1186/s12870-026-09654-9

**Published:** 2026-08-03

**Authors:** Mikias D. Guche, Lorenza Dalla Costa, Alessandra Lanubile, Axel Mithöfer, Domenico Masuero, Francesco Trenti, Mar Garcia-Aloy, Silvia Vezzulli, Urska Vrhovsek, Graziano Guella, Mickael Malnoy, Adriano Marocco, Stefania Pilati

**Affiliations:** 1 C3A - Center Agriculture Food Environment, University of Trento, Via Edmund Mach 1, San Michele all’Adige, 38098 Italy; 2 Research and Innovation Centre, Fondazione Edmund Mach, Via E. Mach 1, San Michele all’Adige, 38098 Italy; 3 Department of Sustainable Crop Production, Università Cattolica del Sacro Cuore, Via Emilia Parmense 84, Piacenza, 29122 Italy; 4 Research Group Plant Defense Physiology, Max-Planck-Institute for Chemical Ecology, Hans-Knöll-Str. 8, Jena, D-07745 Germany; 5 Department of Physics, University of Trento, Via Sommarive 14, Povo, 38123 Italy

**Keywords:** Vitis spp., Powdery mildew, Lipoxygenases, Lipidomic, Salicylic acid, Defensin

## Abstract

**Background:**

Powdery mildew, caused by the obligate biotroph *Erysiphe necator*, represents a major threat to grapevine production worldwide. Host-mediated resistance offers a sustainable alternative to chemical fungicides. To elucidate the role of plant membrane lipid modifications and oxylipin accumulation in pathogen perception and response, a controlled infection experiment was conducted comparing a resistant hybrid (NY_39) from the Edmund Mach Foundation germplasm collection with a susceptible variety (cv. ‘Teroldego’).

**Results:**

A lipidomic approach was integrated with hormone profiling and lipoxygenase (LOX) gene expression analysis. Significant lipid modulation was observed following *E. necator* inoculation, highlighting the plasticity of membrane and storage lipids metabolism. A rapid decrease in triacylglycerol content was measured in NY_39 at 12 h post-inoculation (hpi), whereas the opposite trend occurred in ‘Teroldego’, suggesting divergent metabolic rearrangements. A fast galactolipids peroxidation occurred in NY_39, while a delayed accumulation of phosphatidic acid was observed in ‘Teroldego’ at 48 hpi. The resistant genotype exhibited higher constitutive levels of salicylic acid as well as the expression of the *VviAMP1* defensin, a small cysteine-rich protein with broad-spectrum antifungal activity. *VviPR10-s11* and *VviWRKY51*, related to the synthesis of lignin and stilbenoid phytoalexins, were induced at 12 hpi in both genotypes. Within the LOX family, significant up-regulation of the 9-LOX *VviLOX1a* and the 13-LOXs *VviLOX9* and *VviLOXO* was specific to ‘Teroldego’ at 12 hpi, despite a higher constitutive level of *VviLOX9* in NY_39.

**Conclusions:**

This work identifies key metabolic, signalling and gene expression features in the selected hybrid that may be relevant for resistance. These include rapid triacylglycerols degradation, galactolipid peroxidation, constitutive higher SA level and expression of the defensin *VviAMP1*.

**Supplementary Information:**

The online version contains supplementary material available at https://doi.org/10.1186/s12870-026-09654-9.

## Introduction

Powdery mildew (PM) is one of the most relevant diseases in viticulture worldwide, particularly harmful for the cultivated Eurasian grapevine, *Vitis vinifera* L [1, 2]. Its causal agent, *Erysiphe necator* (Schw. anamorph *Oidium tuckeri* Berk.), belongs to the Ascomycota family and is an obligate biotroph infecting all green parts of the plant. In highly humid environments, infection pressure can be so high as to heavily reduce grape yield and quality [3]. Fungicides and inorganic substances, above all sulfur, are widely used to control this disease, but they present several drawbacks, such as high costs, threats to human and environmental health and the emergence of fungicide resistance in *E. necator* populations [4, 5]. Breeding strategies for durable PM resistance in grapevine cultivars represent a more sustainable approach for grape and wine industries [6, 7].

*Erysiphe necator* infection mechanism relies on the germination of air spread conidiospores on surfaces of plant green tissues producing a lobed appressorium, from which a penetration peg emerges to invade the epidermal cell. A specialized intracellular structure called haustorium facilitates the dynamic exchange of nutrients from host cells and proteins from the fungus, aimed at suppressing host defence. If the infection is established, hyphal growth is observed, with new appressoria and haustoria [2]. To resist pathogens, grapevine has evolved an innate immune system based on the recognition of pathogen-associated molecular patterns called PTI (PAMP-triggered immunity) and a race-cultivar-specific response, known as ETI (effector-triggered immunity), initiated by pathogen virulence factors and mediated by resistance (*R*) genes, to activate the programmed cell death (PCD) [2, 8, 9]. So far, 12 different *Ren* (*E. necator*) and two *Run* (former, *Uncinula necator*) resistance loci have been identified in wild North American grapevine species (*Muscadinia rotundifolia* (syn. *Vitis rotundifolia*), *V. rupestris*, *V. riparia*, *V. aestivalis*, *V. labrusca*, and *V. berlandieri*) as well as wild Chinese species (*V. romanetii* and *V. piasezkii*) [10]. A strategy to achieve long-lasting PM resistance relies on the stacking of these *R*-loci, a crossbreeding process named *R* gene “pyramiding”. However, the signalling mechanisms and metabolic changes related to resistance in these genotypes are so far poorly described.

Plant hormones, mainly salicylic acid (SA), ethylene and jasmonic acid (JA), modulate defence-related pathways, including oxidative burst, cell wall reinforcement, biosynthesis of antimicrobial phytoalexins and accumulation of pathogenesis-related (PR) proteins [11, 12]. The antagonistic model of SA and JA-mediated pathways against biotrophs and necrotrophs, respectively, initially described in *Arabidopsis* [13] has been challenged in many later studies, highlighting the specificity of each pathosystem. For example, in poplar infected with the biotroph *Melampsora larici-populina*, SA and JA interacted positively to stimulate the accumulation of flavonoid phytoalexins [14]. In *Arabidopsis*, a spatially resolved study of the interaction with *Pseudomonas syringae* pv. *tomato* DC3000 highlighted the requirement of both SA and JA for ETI, activating different genes in different areas around the infection site [15]. In grapevine, the role of SA in PCD stimulation during ETI has been assessed, while the role of JA has been reported in response to the oomycetes *Plasmopara viticola* [16, 17]. Lipids- as reservoirs of JA and oxylipins precursors [18, 19], physical barrier and carbon storage - represent crucial components in the plant-pathogen interactions in both PTI [20] and ETI [21]. Changes in free fatty acids, oxylipins and jasmonates content were previously reported in several pathosystems [22–26]. In grapevine, lipid modulation has been studied during the interaction with *Plasmopara viticola* and *E. necator*, in resistant and susceptible varieties. The modulation of lipids and the expression of phospholipase and desaturase genes during early interaction with *P. viticola* highlighted a relevant role of chloroplast membranes, whose fluidity and plasticity were affected, together with changes in the free fatty acid content, accumulation of JA and its conjugated form with isoleucine (JA-Ile) and modulation of JA-related genes [27–31]. Regarding the interaction with *E. necator*, a metabolomic study considering three resistant genotypes (two mono-locus and one with two *R*-loci) and a susceptible one highlighted enhanced levels of four free fatty acids (behenic acid, palmitoleic acid, arachidic acid, and oleic acid + *cis* vaccenic) and one triterpenoid (oleanolic acid) in resistant grapevine varieties in response to the pathogen [32]. A dedicated lipidomic study on three pyramided and one susceptible genotypes revealed a prevalent reduction in di-galactolipid and phosphatidic acid content at 24 h post inoculation (hpi) in the two highly resistant genotypes and later, more extensive and opposite modulation in the susceptible varieties [33].

Lipoxygenases (LOXs) catalyse the peroxidation of both free polyunsaturated fatty acids (PUFAs, such as linoleic, linolenic and hexadecatrienoic acids) and esterified lipids such as galactolipids and triacylglycerols (TG) [23, 34]. Peroxidized products are further metabolized by the enzymes of the seven major branches of LOX pathway [35]; in grapevine, the AOS and HPL pathways have been studied. The gene *VviAOS*, encoding a chloroplastic enzyme, is induced by wounding and contributes to JA biosynthesis [36]. Two HPL genes have also been identified: *VviHPL1*, encoding a chloroplastic enzyme responsible for producing C6 aldehydes, and *VviHPL2*, encoding a cytosolic isoform that releases C6 and C9 aldehydes [37, 38]. The two chloroplastic AOS and HPL enzymes likely compete for the substrate, antagonistically stimulating either the jasmonate-dependent basal immunity (PTI) or participating in the signalling of cell-death related defence (PCD) [37]. In the grapevine genome release available in 2010 (12X.V0), 12 *LOX* genes were identified, three of which with predicted 9-LOX activity and the remaining 9 with 13-LOX activity [39]. Reports on the involvement of *VviLOX* genes in defence against biotic stress are limited. Podolyan and co-workers (2010) observed a rapid induction of *VvLOXC* and -*O* upon mechanical wounding and inoculation of berries with *Botrytis cinerea. VviLOXA*, together with *VviAOS* and *VviOPR2*, were induced by PM infection and SA treatment in the susceptible cultivar (cv.) ‘Cabernet Sauvignon’ [40]. A 9-*LOX* (*Vitvi14g00234*) was induced by *P. viticola* inoculation, and also by the biocontrol agent *Trichoderma harzianum T39*, which confers tolerance possibly by activating genes related to JA and ethylene signalling [41].

The aim of this study was to gain insight into the role of SA and JA in the interaction between grapevine and the biotrophic ascomycete *E. necator* in resistant and susceptible genotypes, as well as the role played by lipids both in pathogen perception and signalling and as precursors of JA, whose biosynthesis might be related to the transcriptional up-regulation of specific *LOX* isoforms. The identification of molecular mechanisms and genes relevant for pathogen resistance will support new breeding strategies.

## Materials and methods

### Plant material

Woody cuttings from the PM susceptible *V. vinifera* cv. ‘Teroldego’ and the PM resistant *Vitis* hybrid NY_39 were collected at the germplasm collection maintained at Fondazione Edmund Mach (FEM) (Trentino-Alto Adige, Italy). The resistant breeding selection derived from the cross between NY95.0308.02 (♀; *Ren2* donor) and Eger 99-11.01 (♂; *Run1*, *Ren3* and *Ren9* donor) made by USDA (Geneva, NY, USA) to stack (“pyramid”) multiple *R*-loci conferring *E. necator* resistance into the same individual. In this framework, the F_1_ individual NY_39 was selected because pyramided for *Run1*, *Ren2*, *Ren3*, and *Ren9* loci and thus valuable as a superior parent in crossbreeding programs. Cold hardened cuttings were treated with fungicide and grown on sterilized media (Jiffy pellets) in a growth chamber under controlled (16/8 h photoperiod and 24 ± 2 °C temperature) conditions. Rooted cuttings were potted and transferred to the greenhouse, for 5 weeks. In the sixth week, plants were transferred to the infection chamber, for a 2-weeks acclimation. Three treatments with cyflufenamid 5.1% (Cidely) at 400 µl/L H_2_O were performed at 2, 4, and 6 weeks (the last one in the infection chamber) after bud burst to avoid uncontrolled PM infections.

### *Erysiphe necator* inoculation assay

*Erysiphe necator* inoculum consisted of a variety of strains sourced from naturally infected leaves of *V. vinifera* plants and propagated in vitro on detached leaves, following the dry method [32]. A total of 12 randomly selected plants from each genotype were used for inoculation assay and each plant was considered as a biological replicate. *E. necator* inoculation experiment was carried out 8 weeks after bud burst in the infection chamber (22 ± 2 °C and 80% RH under natural light conditions). Inoculation was made in the morning (6:30 to 7:00 AM) on whole plants, on fully opened second and third leaves using 2 weeks old in vitro-maintained conidia by gently tapping a sporulating infected leaf above the adaxial leaf surface. Control (uninoculated leaves, time zero) and inoculated leaves at 12 (7 PM), 24 (7 AM), 48 hpi (7 AM) were collected in three replications and samples were immediately frozen in liquid nitrogen and stored at -80 °C for gene expression, lipidomic and phytohormonal analysis. At the same time, inoculated leaf samples from corresponding plants were collected and processed immediately for microscopic evaluation at 12, 24 and 48 hpi.

### Microscopy assessment

To evaluate the fungal structures, 25 mm diameter leaf discs were excised (adaxial surface up) and fixed according to [42] and stained with aniline blue (0.1%, v/v) in lactoglycerol immediately before microscopic observation. Microscopic images for 12hpi were captured using Leica LMD700 compound microscope at 200X magnification, while for 24 and 48 hpi observations were conducted using Leica MZ16 stereomicroscope at 100X magnification. *E. necator* establishment was semi-quantitatively evaluated by counting successful appressorium formation at 12 hpi from a 100–150 randomly taken conidia and counts were expressed as percentage over total conidia. In addition, the formation of successful colonies was evaluated in the same way by counting the percentage of established conidia with secondary hyphal formation in different areas of the leaf. Secondary hyphae length was measured from captured images of leaf discs using the image processor software ImageJ [43].

### Powdery mildew resistance evaluation

Powdery mildew symptoms on the second and third leaves of each shoot were evaluated at 15 days post inoculation (dpi) using the 5-class rating system according to the International Organisation of Vine and Wine (OIV) descriptor 455 for “degree of resistance to *Oidium*” [10]: 1 - unlimited infection with ample mycelium and fungus fructification, 3 - vast attacked patches and obvious mycelial growth and fungus fructification, 5 - disease patches with 2–5 cm diameter, 7 - patches with 2 cm or less diameter with little mycelium and limited fungus fructification and 9 – suppressed or no symptoms and mycelium or visible fructification.

### Lipidomic analysis

Total lipids were extracted as described in [33, 44]. Briefly, two extractions were made from 100 mg of frozen leaf samples in three biological replicates. To each sample, 300 µL of methanol (CH_3_OH) and 600 µL of chloroform (ClCH_3_) containing butylated hydroxytoluene (BHT 500 mg/L) and triphenylphosphine (TPP, 260 mg/L), and 15 µL of internal standards (IS) mix (0.5 mg/L of each standard, listed in Suppl. Table S1), were added. After gently shaking for 60 min, 250 µL of Milli-Q purified H_2_O were added and the extracting mixture was centrifuged at 3600 rpm for 10 min at 4 ◦C. The total lipid-rich layer was transferred to a new microtube. For the second extraction, 400 µL of CHCl_3_/CH_3_OH/H_2_O 86:13:1 (v/v/v) was used, followed by centrifugation, extraction, and the lower lipid phase was added to the first lipid fraction. Solvent was evaporated under a stream of nitrogen, and the dried extracts were reconstituted in 300 µL of acetonitrile/isopropanol/water (65/35/5). For semi-quantitative targeted lipidomic, the liquid chromatography separation was performed with an Exion LC system provided by AB Sciex LLC (Framingham, MA, USA) coupled with an AB Sciex QTRAP 6500+ (Framingham, MA, USA) mass spectrometer. The identification and quantification of the lipids was done according to [44]. The device was run in Multiple Reaction Monitoring (MRM) mode with electrospray ionization (ESI) voltage set at 5500 and − 4500 V for positive and negative mode, respectively. Spectral data was processed using MultiQuant, version 3.0 (Sciex, Concord, Vaughan, ON, Canada) and quantification was calculated using one internal standard for each class.

Untargeted lipid peroxidation analysis was performed on the same samples used for the semi-quantitative analysis applying the protocol described in [23]. Briefly, the lipid extract was analyzed by liquid chromatography-mass spectrometry (LC- MS) (Model 1100 series; Hewlett-Packard; Palo Alto, CA, USA) coupled to a quadrupole ion-trap mass spectrometer (Esquire LCTM; Bruker, Bremen, Germany) equipped with an electrospray ionization source. Chromatographic separation of lipids was carried out at 303 K using a C18 column (TMC18; length, 100 mm; particle size, 2.6 μm; internal diameter, 2.1 mm; pore size, 100 Å; Phenomenex, Torrence, CA, USA). The solvent system consisted of eluant A as MeOH/H_2_O (7:3, v/v) containing 10 mM ammonium acetate and eluant B as isopropanol/MeOH (10:90, v/v) containing 10 mM ammonium acetate. Samples were resuspended in MeOH/CHCl_3_ (4:1, v/v) to a final concentration of 1 mg/mL, and 10 µL were run with a linear gradient from 65% eluant B to 100% in 40 min, plus 20 min of isocratic 100% B at 1 mL/min to elute the diglycerides and triglycerides. The column was then equilibrated to 65% eluant B for 10 min. The MS scan range was 13,000 U/s in the range from 50 to 1500 m/z, with a mass accuracy of ~ 100 ppm. The nebulizer gas was high purity nitrogen at a pressure range of 20–30 psi, at a flow rate of 6 L/min, and at 300 ◦C. The electrospray ionization (ESI) was operated in positive ion mode for the analysis of galactolipids, phosphatidylcholine (PC) and triglycerides (TG) and in negative mode for chain identification. Data was analysed with the Data Analysis software (Agilent). To verify the occurrence of lipid peroxidation, TG 54:9, TAG 54:6, TAG 54:5, phosphatidylcholines (PC) 36:6, PC 36:5, PC 36:3, di-galactolipids (DGDG) 36:6 and mono-galactolipids (MGDG) 36:6 and the corresponding hydroxylated forms (resulting from TPP reduction of hydroperoxyl groups) were searched as extracted ion chromatograms (EIC) (Suppl. Table S2). For TG and PC, no hydroxylated forms were identified, while for DGDG and MGDG mono and di-peroxidized forms were found. The relative percentage of peroxidation of DGDG, MGDG was calculated as the ratio of the absolute peak area of the EIC of each oxidized species with respect to the peak area of the EIC of total (native and oxidized) corresponding galactolipid species.

### Phytohormonal analysis

Phytohormone extraction was carried out according to [45] from 25 mg (dry weight) of frozen, ground cryo-lyophilized control (not inoculated leaves) and *E. necator* inoculated (12 and 24 hpi) leaf sample from ‘Teroldego’ and NY_39. Five biological replicates were analyzed, 2 of them obtained from additional plants grown to ensure sufficient tissue availability. Phytohormone analysis was performed by LC-MS/MS using previously described methods [46, 47] on an Agilent 1260 series HPLC system (Agilent Technologies) coupled to a tandem mass spectrometer QTRAP 6500 (SCIEX, Darmstadt, Germany).

### RNA isolation and gene expression analysis

Total RNA was extracted from 100 mg of powdered leaf samples using Spectrum™ Plant Total RNA kit (Sigma-Aldrich, St Louis, MO, USA), treated with Amplification Grade DNase I (AMPD1, Sigma-Aldrich, St Louis, MO, USA) and quantified using Nanodrop 8800 (Thermo Fisher Scientific, USA). RNA integrity was checked by gel electrophoresis. cDNA was reverse transcribed from 2 µg of total RNA using SuperScript™ III (Thermo Fisher Scientific, USA). Quantitative PCR (qPCR) reactions were carried out in a 12 µl reaction volume containing 2 µl 1:10 diluted first strand cDNA, 1X SSOFast EvaGreen supermix (Bio-Rad, Hercules, CA, USA) and forward and reverse primers at the final concentration of 300 nM. PCR reactions were run using Bio-Rad CFX96™ Real-Time PCR System (Bio-Rad, Hercules, CA, USA) with cycling conditions of a 95 °C for 3 min, 40 cycles of denaturation (95 °C for 15 s), annealing/extension (primer specific annealing temperature for 30 s) and melting curve analysis. Two sets of specific primers were designed for each of the ten *LOX* genes using the NCBI Primer-BLAST tool and a preliminary assay was carried out to evaluate their specificity (Supplementary Fig. 1). The two sets of primers amplified regions of different sizes, one around 100–120 bp (short) and the other around 150–170 bp (long). Two cDNA templates were used, derived from RNA extracted from the leaves of ‘Sugraone’ plants cultivated in the greenhouse and in the field, respectively. The selected primers are listed in Suppl. Table 3. *Vitis vinifera* actin [48] and glyceraldehyde 3-phosphate dehydrogenase [49] were used as housekeeping genes for normalization. The relative quantification of gene expression was calculated with the method proposed by [50], using two technical replicates for each of the three biological replicates. The PCR efficiency of each reaction was obtained using the LinRegPCR program version 2014.x [51] and the normalized relative quantity (NRQ) were calculated over all samples with the 2^−ΔΔ^Ct method.

### Phylogenetic analysis

Lipoxygenase protein sequences were retrieved by HMMER (version 3.3.2) [52] search based on the Pfam domains PF01477 and PF00305 using the grapevine *V. vinifera* cv. PN40024, VCost.v3 annotation of the 12X.v2 genome assembly [53] and *Arabidopsis thaliana* version TAIR 10.1 [54]. The grapevine lipoxygenase sequences were further curated manually using the Apollo platform [55].

Multiple sequence alignment of the 19 amino acid sequences was carried out using MUSCLE [56] and the best-fit amino acid substitution model was determined using the maximum likelihood (ML) estimation approach in MEGA X [57] and the substitution model with the lowest BIC score (Bayesian Information Criterion) was considered for phylogenetic analysis. *De novo* phylogenetic tree was constructed using the Maximum Likelihood method MEGA X using [58] amino acid substitution matrix with gamma distribution (5 categories (+ G, parameter = 1.5152)) allowing evolutionarily invariable ([+ I], 10.01% sites) and the tree with the highest log likelihood was selected. Initial tree(s) for the heuristic search were obtained by Neighbour-Join and BioNJ algorithms using the JTT model. All positions containing gaps and missing data were eliminated (complete deletion) retaining a total of 744 positions for the analysis. A bootstrap test (1000 replicates) was conducted to determine the branch support level for each associated taxon clustered. *De novo* phylogenetic tree was used to establish *Arabidopsis* orthologs *LOXs* to assign *Arabidopsis*-like nomenclature of the grapevine *LOXs* following the recommended gene naming system [59]. The presence of N-term chloroplastic peptide was predicted using TargetP ver 2.0 [60].

To further validate the conservation/evolutionary pattern between *V. vinifera* and *Arabidopsis LOXs*, a synteny analysis in TBtools was conducted [61]. Genome and annotation files previously used for the phylogenetic analysis were retrieved and one-step MCScanX tool [62] was employed to compare the sequence against itself and other genomes with BLASTP parameters of E-value threshold of 1e^− 5^ and best 5 non-self-hits.

### Gene network analysis

Genes modulated by PM in a salicylic acid (SA)-dependent or independent manner were retrieved from Toth et al. (2016) (Supplementary Dataset 1). These gene sets were intersected with the *VviWRKY51* and *VviPR10-s11* OneGenE lists of associated genes [63], that can be retrieved from the web-tool OneGenE Causality Weaver (https://onegene-causality-weaver.disi.unitn.it/vitis/downloads/), section called “Download of specific expansion lists”. The final list formed by the 55 genes identified as PM responsive and associated to *VviWRKY51* and *VviPR10-s11* has been used as a query (https://onegene-causality-weaver.disi.unitn.it/vitis2/network/) to reconstruct the gene network, and visualize their causal relationships. The network, formed by 55 nodes and 405 edges, was exported as .csv text file and imported into Cytoscape [64], where node colour and shape was customized and node labelled improved by integrating the available functional annotation, where the gene symbol was not assigned. “Unknown” genes were left unlabelled and not visualized.

### Statistical analysis

Lipid semi-quantitative data (ng/mg) and relative peroxidation data was analysed with R software (version 4.5.2). Effect size was used to identify lipids with clear differences between groups. The Cohens’d method from the R package “effectsize” (version 1.0.2) was applied on log10-transformed data and an absolute value > 1 was used as a. The statistical significance of the treatment effect was evaluated by fitting a linear model for each genotype on log10-transformed data. The p-values for pairwise comparisons between consecutive time points were calculated by estimated marginal means using the package “emmeans” (version 2.0.2). Then, principal component analysis (PCA) using log10-transformed and scaled data was performed to get an overview of the data. One sample of the ‘Teroldego’ genotype at 48 hpi was excluded because it had missing values for all mono- and di-galactolipids (MGDG and DGDG).

Hormone and gene expression data were analysed with R software (version 4.6.0). For hormone-related compounds, five biological replicates were employed, and data were log2 transformed. The normal distribution of the interactive two-way ANOVA residuals was confirmed using the Shapiro-Wilk test, and Tukey’s Honest Significant Difference (HSD) post-hoc test was applied to evaluate significant differences among genotypes and time-points. Similarly, for the gene expression data, the log2-transformed normalized relative quantities (NRQs) were first evaluated for residual normality. Tukey’s HSD multiple comparison procedure was then applied exclusively to the 14 genes (out of 17) that successfully passed the Shapiro-Wilk test, whereas the remaining 3 genes (*VviLOXA*, *VviLOX14* and *VviNPR1*) were treated descriptively. To evaluate PM resistance of the two genotypes using the OIV-455 scoring scale, a nonparametric Mann-Whitney test was used while to compare fungal secondary hyphal growth on the leaves of the two genotypes a nonparametric Welch’s t-test was applied, using BioRender (Biorender.com).

## Results

### The pyramided genotype showed complete and fast post-penetration resistance to *E. necator*

**Fig. 1:**
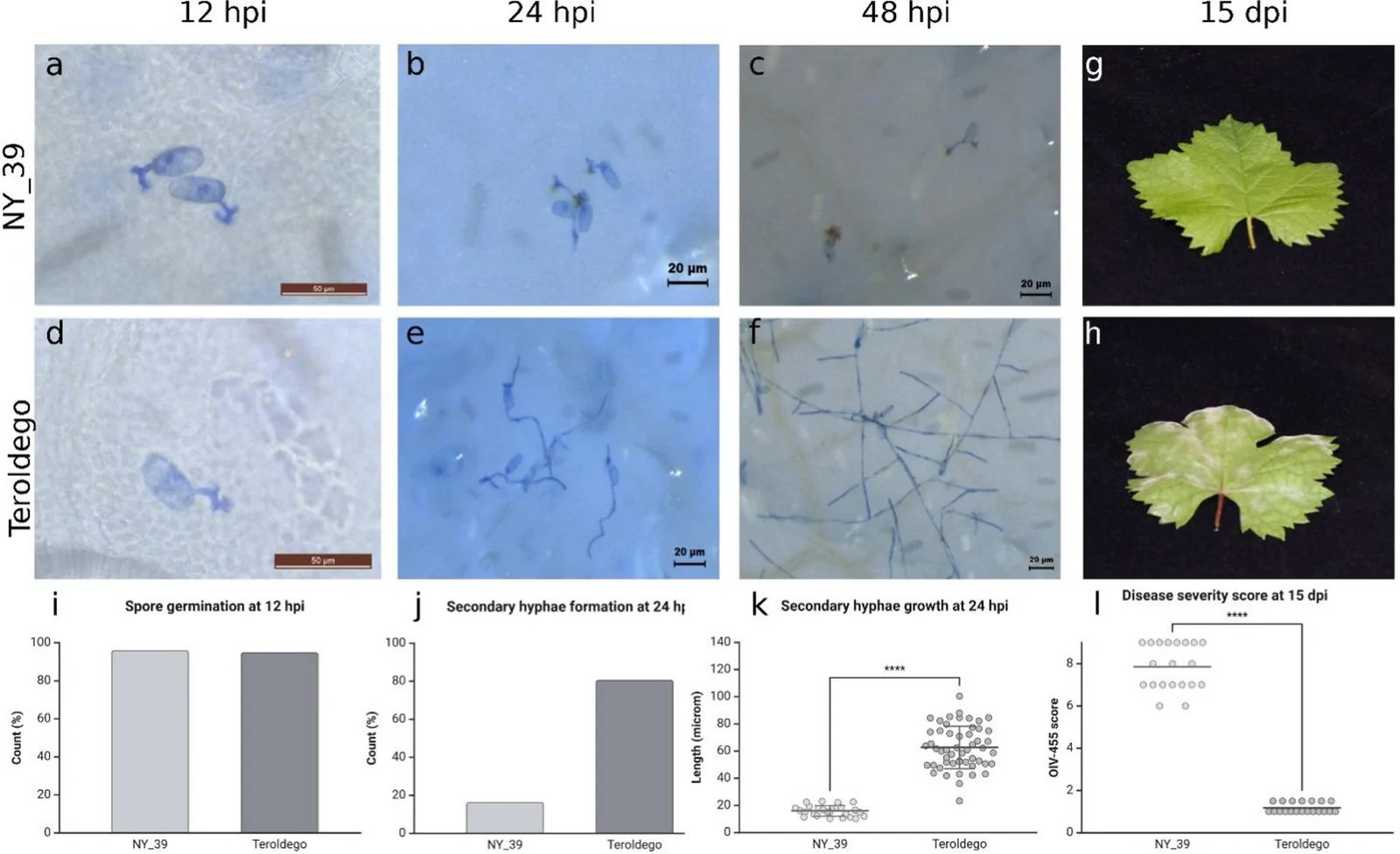
Microscopic assessment of the early interaction between *E. necator* and the resistant NY_39 genotype and the susceptible *V. vinifera* cv. ‘Teroldego’ and resistance evaluation after 15 days. **a-f**: Images of fungal structures were taken from adaxial surfaces of inoculated leaf discs at 12, 24 and 48 hpi, fixed and stained with 0.1% (w/v) aniline blue. **g** Suppressed or no symptoms and mycelium at 15 dpi on NY_39. **h** Extensive mycelial growth and conidiophore production at 15 dpi on ‘Teroldego’. **i** Percentage of conidiospore germination estimated by counting primary appressorium formation (100–150 conidiospores). **j** Secondary hypha formation at 24 hpi estimated by evaluating 100–150 conidia. **k** Secondary hyphae growth estimated measuring hyphal length at 24 hpi: dots represent replicates (*n* = 54 for Teroldego, *n* = 26 for NY_39), mean is reported with standard deviation (SD) represented as whiskers, significance was estimated using nonparametric Welch’s two tailed t-test and **** indicate significant difference (*p* ≤ 0.0001). **l** PM disease severity evaluated at 15 dpi, using the OIV-455 descriptor ranging from 1 (very low) to 9 (very high or complete resistance); dots represent replicates (*n* = 20), mean is reported with standard deviation (SD) represented as whiskers. Significance was estimated using non-parametric Mann-Whitney test and **** indicate significant difference (*p* ≤ 0.0001)

To investigate the PM resistance mechanism, artificial infection with *E. necator* conidia was conducted on young leaves of the resistant hybrid NY_39 and the susceptible *V. vinifera* cv. ‘Teroldego’ plants under controlled conditions in the greenhouse. Microscopic and macroscopic inspections are displayed in (Fig. 1).

At 12 hpi, 95% and 96% of *E. necator* conidia germinated producing primary appressorium on both NY_39 and ‘Teroldego’ genotypes, respectively (Fig. 1a, d, i). However, the rate of successful post-penetration conidial establishment, measured as secondary hyphal formation, was highly restricted in the pyramided genotype NY_39 (16,5%) already at 24 hpi, limiting subsequent hyphal proliferation (Fig. 1b, j). Conversely, a higher rate of compatible interaction was observed in the susceptible genotype with 81% of the germinated conidia forming secondary hyphae at 24 hpi (Fig. 1e, j). Quantitative evaluation of secondary hyphal length at 24 hpi (Fig. 1k) confirmed the same trend, being significantly lower in the NY_39 (15.91 ± 0.80 μm) respect to the susceptible genotype (62.64 ± 2.10 μm). Moreover, typical hypersensitive reactions were observed at the pathogen penetration site in NY_39 at 24 hpi (Fig. 1b) with evident browning and necrotic regions, typical of PCD, which were totally absent in ‘Teroldego’ (Fig. 1e). Even at 48 hpi, *E. necator* progression was not evident in NY_39, while fungal proliferation showed a robust increase in ‘Teroldego’, where primary hyphal branches were successfully formed at a rate of 3 to 5 branches per single colony forming conidia (Fig. 1c, f). The resistance level of the genotypes was also characterized at 15 dpi according to the adapted OIV-455 descriptor (indirect severity index), which ranges from 1 (very low resistance) to 9 (very high or complete resistance). NY_39 leaves showed no visible macro symptoms and tiny patches of necrotic/yellow spots and mycelial and conidiophore structures were absent in all evaluated leaves, corresponding to high scores of resistance (Fig. 1g, l). In contrast, ‘Teroldego’ showed vast patches of *E. necator* colonies with abundant mycelial growth and strong sporulation, corresponding to very high susceptibility (and very low OIV-455 score) (Fig. 1h, l).

### Resistant and susceptible genotypes showed different lipid modulation upon E. necator inoculation

**Fig. 2:**
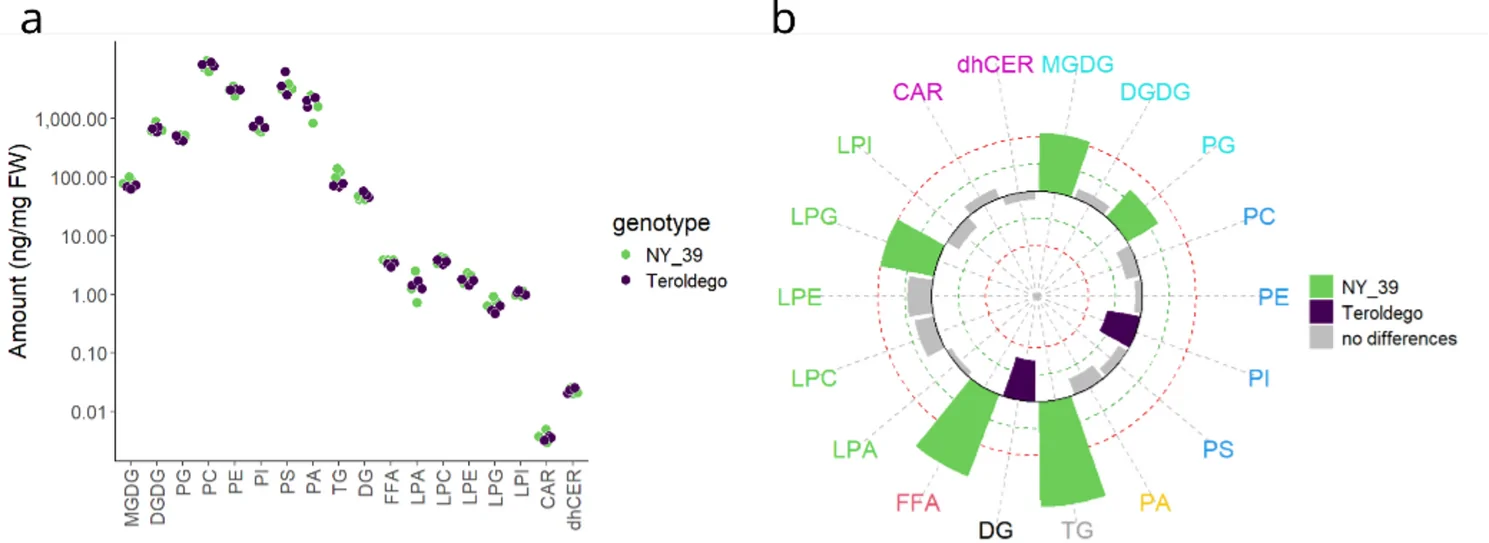
Overview of the lipid composition of leaf samples taken from NY_39 and ‘Teroldego’ plants before *E. necator *inoculation.** a** Overall abundance of each lipid class. The sum of the amount of each species within a class is reported as dots for each biological replicate for each genotype. The amount is reported as ng/mg FW using a log10 scale. **b** Comparison between the two grapevine genotypes. Circular plot showing the effect size (ES) values obtained when comparing the total amount of quantified species for each lipid class in each genotype. Circular lines colours black, green and red represent values of effect size 0, +/- 1, and +/- 2, respectively. Classes are coloured according to functional categories, green and purple outwards or inward projected barplots indicate classes with absolute effect size > 1, for NY_39 and ‘Teroldego’, respectively. CAR: carnitine; DG: diacylglycerol; DGDG: digalactosyldiacylglycerol; dhCER: dihydroceramide; FFA: free fatty acid; LPA: lysophosphatidic acid; LPC: lysophosphatidylcholine; LPE: lysophosphatidylethanolamine; LPI: lysophosphatidylinositol; LPG: lysophosphatidylglycerol; MGDG: monogalactosyldiacylglycerol; PA: phosphatidic acid; PC: phosphatidylcholine; PE: phosphatidylethanolamine; PI: phosphatidylinositol; PG: phosphatidylglycerol; PS: phosphatidylserine; TG: triacylglycerol

The lipidomic profile of uninoculated (control) and *E. necator* inoculated leaves of NY_39 and ‘Teroldego’ sampled at 12, 24 and 48 hpi covered 287 different compounds, grouped into 18 classes (Suppl. Tables 4–5). An initial analysis of the control samples of the two genotypes was carried out to gain an overview on cell lipid composition and estimate their basal, constitutive diversity (Fig. 2, Suppl. Figure 2). Cell membrane phospholipids, such as PC, PE, PS and PA were the most abundant (in the range of micrograms per mg FW) classes for both genotypes, followed by phosphatidyl-inositols (PI), digalactolipids (DGDG) and phosphatidylglycerols (PG) (Fig. 2a). Monogalactolipids (MGDG), storage lipids (TG) and lipid precursors (diacylglycerols, DG) were also abundant, but to a lower extent (in the range of 100 nanograms per mg FW). Free fatty acids (FFA) and lysophospholipids were in the range of nanograms per mg FW, while the less abundant species were ceramide and carnitines. These lipidomic profiles are in line with those reported for young leaves of *Arabidopsis* and tobacco, characterized by lower amounts of TG, DG and galactolipids and higher amounts of phospholipids, when compared to older leaves [65, 66]. According to Cohen’s effect size index, TG and FFA (18:2 and 18:1 in particular) as well as chloroplast membrane constituents MGDG, PG and its partially hydrolysed form lyso-phosphatidylglycerol (lysoPG) were more abundant in NY_39 compared to ‘Teroldego’ (Fig. 2b and Suppl. Figure 2). Conversely, in ‘Teroldego’ DG and phosphatidylinositol species (PI) were more abundant.

**Fig. 3:**
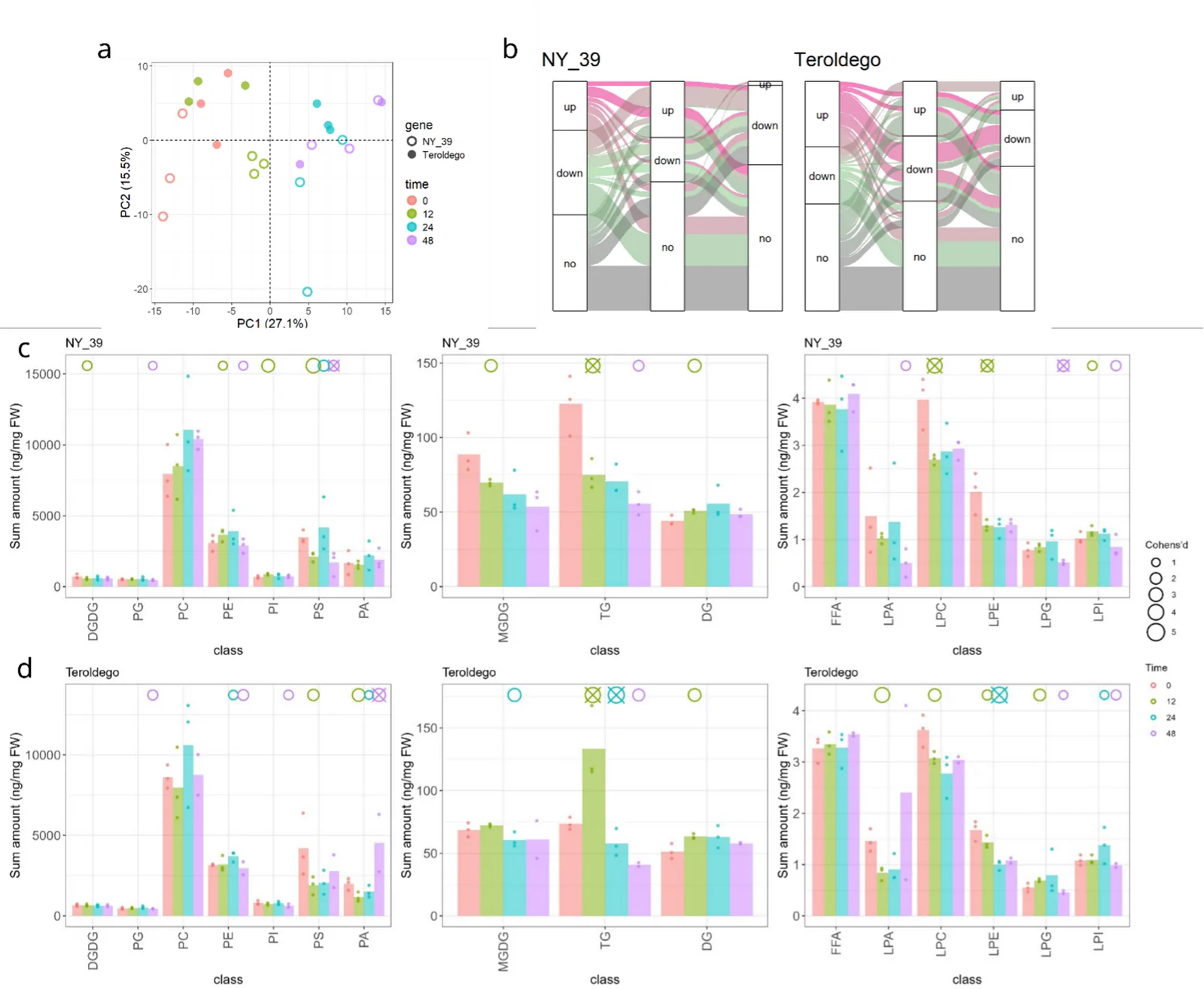
Overview of the lipid modulation after *E. necator *inoculation. **a** PCA analysis of the lipidomic dataset at the different time points after inoculation in the two genotypes. **b** Sankey plot of the lipid species comparing one time-point versus the previous one. Modulation is estimated by computing the effect size for each lipid species between two consecutive time points for each genotype separately; positive and negative modulation refers to a minimum threshold value of + 1/-1, respectively. **c-d** Lipid modulation represented by class in the two genotypes. The sum of the amount of each species within a class is reported as dot for each biological replicate and bars represent the overall average, for each time-point. Empty circles indicate an absolute effect size between each time-point and the previous one higher than 1, with the circle diameter proportional to the magnitude of the effect, whereas crosses indicate those comparisons between consecutive time points reaching statistical significance (*p* < 0.1). The colour of the symbols represents the specific transition: green (0 to 12 hpi), light blue (12hpi to 24 hpi) or violet (24hpi to 48 hpi). DGDG: digalactosyldiacylglycerol; PG: phosphatidylglycerol; PC: phosphatidylcholine; PE: phosphatidylethanolamine; PI: phosphatidylinositol; PS: phosphatidylserine; PA: phosphatidic acid; MGDG: monogalactosyldiacylglycerol; TG: triacylglycerol; DG: diacylglycerol; FFA: free fatty acid; LPA: lysophosphatidic acid; LPC: lysophosphatidylcholine; LPE: lysophosphatidylethanolamine; LPG: lysophosphatidylglycerol; LPI: lysophosphatidylinositol

PCA performed on the entire lipidomic dataset showed that the main source of variability was associated with the time of sample collection after inoculation, separated along the first component which explained 27.1% of the total variance of the dataset (Fig. 3a). Then, genotypes were mostly separated along the second component, explaining 15.5% of the variance.

Sankey plot representation was used to highlight the complexity of lipid modulation within each genotype, focusing only on effect size (Fig. 3b). While effect size cannot be used to assess statistical significance, it serves as an effective exploratory metric to highlight the prevailing trends in lipid accumulation or depletion between consecutive time points. As expected, considering their structural functions, approximately 20% of the lipid species did not exhibit a meaningful effect size for either comparison within both genotypes under the conditions analysed (grey bars at the bottom). Of note, between 24 hpi and 48 hpi, a prevalent negative modulation was observed in NY_39, while in ‘Teroldego’ a more balanced behaviour occurred (see also Suppl. Table 6).

Considering modulation at the lipid class level, effect size and statistical evaluation of the modulation have been considered and visualized (Fig. 3c-d and Suppl. Figure 3). The most contrasting modulation in both terms of the magnitude and statistical evidence of the biological differences was observed for TGs, whose content remarkably increased at 12 hpi in ‘Teroldego’ and decreased in NY_39 (Suppl. Figure 3a). At 24 hpi they decreased also in ‘Teroldego’. Among phospholipids, different trends were observed for phosphatidic acids (PAs), which were modulated only in ‘Teroldego’, with a 2-fold increase at 48 hpi (Suppl. Figure 3d). Other trends, only based on the effect size but not statistically supported, were observed for MGDG galactolipids, whose content decreased at 12 hpi in NY_39 and at 24 hpi in ‘Teroldego’ (Suppl. Figure 3e), and among phospholipids, for PS, for which a transient increase at 24 hpi was observed only in NY_39 (Suppl. Figure 3c). Among the lyso-phospholipids, lyso-phosphatidylcholine (LPC) and lyso-phosphatidylethanolamine (LPE) decreased already at 12 hpi in NY_39, while in ‘Teroldego’ they decreased more gradually. Concerning free fatty acids (FFA), they did not show any significant modulation at the class level (Suppl. Figure 3 g).

**Fig. 4:**
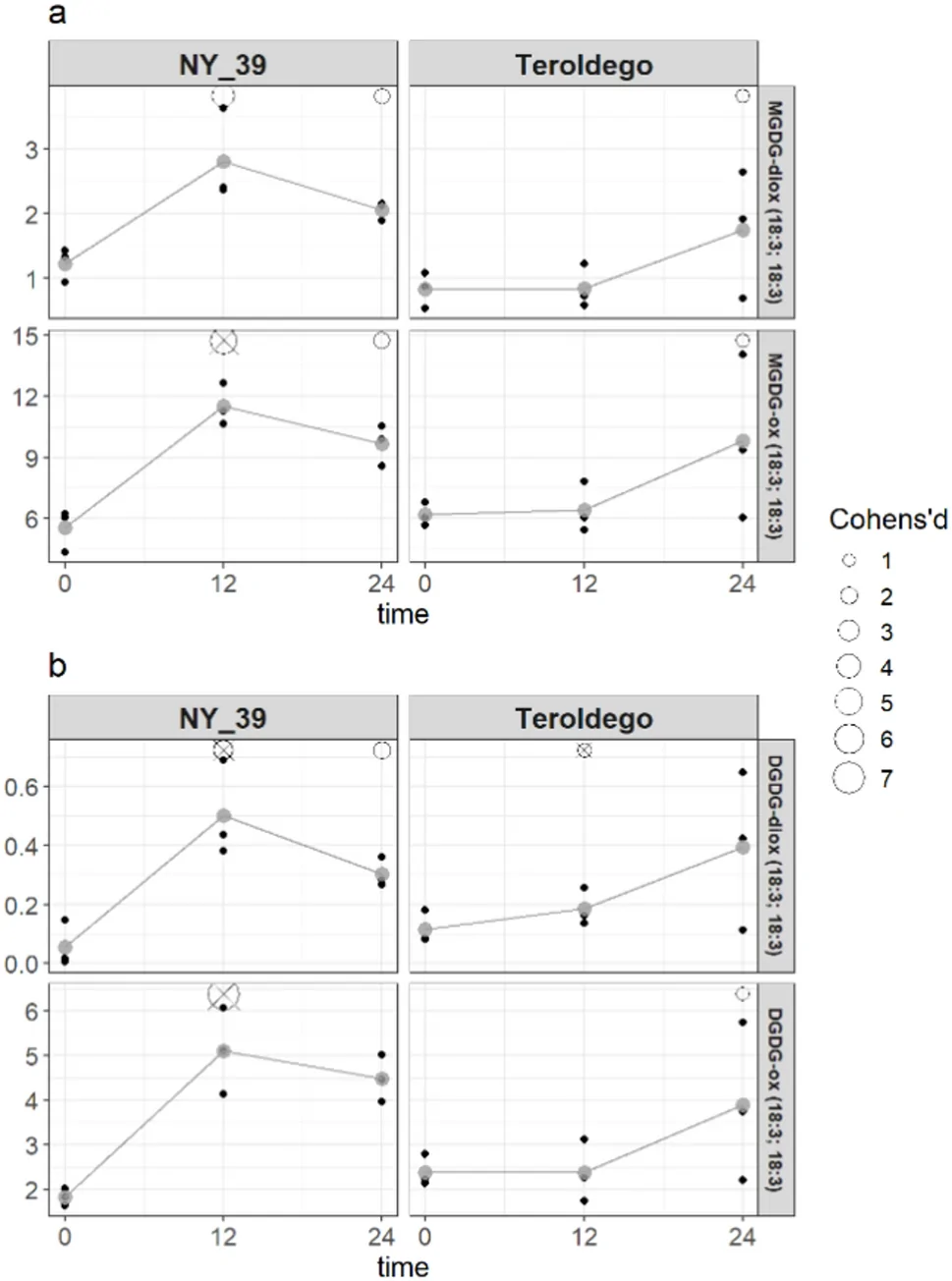
Galactolipid peroxidation after *E. necator* inoculation. Single and double-chain peroxidation of MGDG 36:6 (**a**) and DGDG 36:6 (**b**) is shown at 0, 12 and 24 hpi as relative amounts (%) within each species. Lines connect mean values of each time point. Empty circles indicate an absolute effect size higher than 1 for comparisons between consecutive time points, with the circle diameter proportional to the magnitude of the effect, whereas crosses indicate comparisons that reached statistical significance (*p* < 0.1)

Untargeted metabolomics was applied to study lipid peroxidation. The most abundant PC, TG and galactolipids with poly-unsaturated fatty acid (PUFA) chains were identified, in particular TG 54:9, TG 54:6, TG 54:5, PC 36:6, PC 36:5, PC 36:3, DGDG 36:6 and MGDG 36:6 (Suppl. Table 2). Only for the galactolipid species was it possible to detect the corresponding oxidized species (Fig. 4), suggesting that lipid oxidation occurred inside the chloroplasts and not as a general pattern in the cytoplasm. Despite peroxidation basal levels were not so different in the two genotypes (Suppl. Figure 4), NY_39 showed a rapid accumulation of mono and di-oxidized species of DGDG and mono-oxidized forms of MGDG at 12 hpi, characterized by high values of effect size and statistical significance, whereas in ‘Teroldego’ their increase was much less pronounced in terms of magnitude at both time-points and significant on for di-oxidized forms of DGDG at 12 hpi.

### Powdery mildew resistance correlates with SA increase and defensin expression

**Fig. 5:**
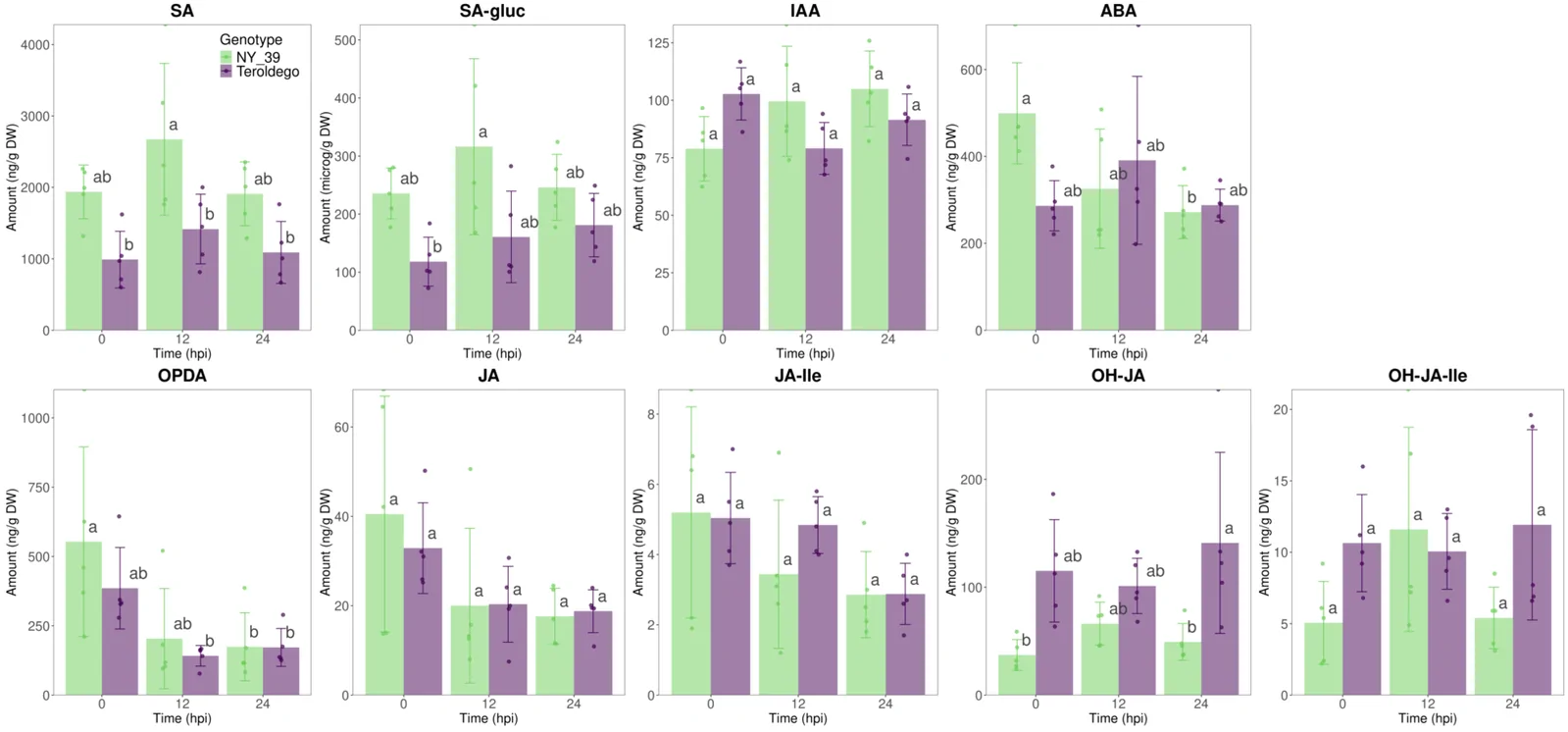
Phytohormonal profile of NY_39 and ‘Teroldego’ leaves inoculated with *E. necator*. Dots represent biological replicates (*n* = 5) while bars and whiskers respectively the mean value and the standard deviation (SD). The amount is reported as ng/g dry weight (DW) using a log2 scale. Statistical significance was assessed using a two-way ANOVA with Tukey multiple comparisons test on log2-transformed data, p-value < 0.05. SA: salicylic acid; SA-Gluc, salicylic glucoside; IAA: indol-3-acetic acid; ABA: abscisic acid; cis-OPDA: 12-oxo-phytodienoic acid; JA: jasmonic acid; JA-Ile: jasmonoyl isoleucine conjugate; 12-OH-JA: 12-hydroxyjasmonic acid; OH-JA-Ile: 12-hydroxyjasmonoyl-isoleucine

Plant phytohormones levels were measured in leaf samples from the resistant hybrid NY_39 and the susceptible ‘Teroldego’ before and after *E. necator* inoculation (Fig. 5). Regarding the SA pathway, NY_39 exhibited a higher basal content of SA and of its storage form salicylic acid glucoside (SA-Gluc) compared to ‘Teroldego’, while no significant changes were measured after pathogen inoculation. Concerning the JA pathway, the precursor *cis*-OPDA significantly decreased at 12 hpi only in NY_39. JA, its conjugated form JA-Ile and its hydroxylated form OH-JA-Ile were not modulated upon inoculation, while the JA hydroxylated form (OH-JA) was significantly lower in NY_39 at both basal level and 24 hpi. Regarding other hormones, Indole-3-acetic acid (IAA) levels were not affected by the pathogen and did not differ significantly between genotypes, while ABA significantly decreased at 24 hpi only in NY_39.

**Fig. 6:**
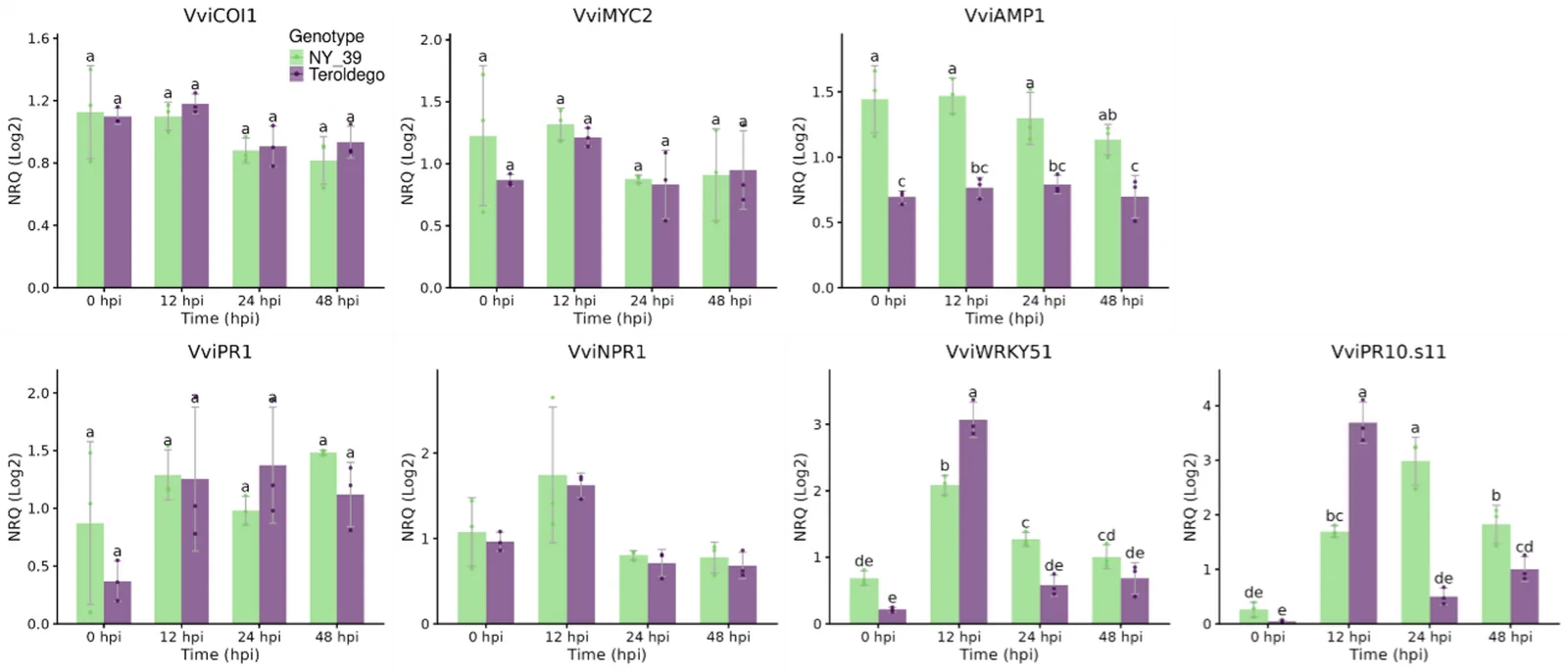
qPCR analysis of grapevine JA and SA pathway-related genes upon *E. necator* inoculation. Expression of selected genes in control and inoculated (12, 24 and 48 hpi) leaves of NY_39 and ‘Teroldego’ is shown. Dots represent biological replicates (*n* = 3), while bars and whiskers respectively the mean value and the standard deviation (SD). Statistical significance was assessed using a two-way ANOVA with Tukey multiple comparisons test on log_2_ transformed normalized relative quantity (NRQ), p-value < 0.05, only for the genes passing the Shapiro-Wilk normality test

Gene expression profiles were analysed for markers associated with both the JA and SA signalling pathways (Fig. 6). For the JA pathway, three genes were tested: *VviCOI1* (*Vitvi13g00512*), the JA-Ile receptor, *VviMYC2* (*Vitvi02g00698*), a transcriptional activator of JA-responsive genes, and *VviAMP1* (*Vitvi18g02760*), a defensin with antifungal activity against *B. cinerea* [67, 68] and orthologous to *Arabidopsis AtPDF1.2* (up-regulated by pathogens via methyl-JA, but not SA, signalling [69]), . *VviCOI1* and *VviMYC2* expression levels were not modulated following pathogen inoculation in either genotype. In contrast, *VviAMP1* displayed a significantly higher basal expression level in the resistant NY_39 background compared to the susceptible ‘Teroldego’; this trend persisted across all post-inoculation time-points, even though the gene itself was not responsive to the pathogen. Regarding the SA signalling pathway, the Non-expressor of PR1 gene (*VviNPR1*, *Vitvi11g00158*), mediator of SA signalling and involved in PM resistance [70], and the *VviPR1* (*Vitvi03g00752*) were analysed. Due to high data variability, no consistent modulation pattern was discernible for *VviPR1*, while the expression data for *VviNPR1* were so fluctuating that parametric statistical inference could not been performed.

Given the lack of dramatic modulation in the core SA and JA marker genes, two additional genes known to be induced during *E. necator* infection were included: the PR protein *VviPR10-s11* (*Vitvi05g01757*), belonging to a gene cluster on chromosome 5 and coding for a protein with nuclease activity, under the control of JA, SA and ABA [71–73], and the transcription factor *VviWRKY51* (*Vitvi16g01213*), previously found induced by PM [74]. Both genes were highly induced in both genotypes: *VviWRKY51* showed an intense transient up-regulation at 12 hpi in both genotypes, while *VviPR10-s11* expression reached a peak at 12 hpi in ‘Teroldego’ and at 24 hpi in NY_39. Finally, a comparative analysis was performed using publicly available transcriptomic data from two studies: one focused on Asian *V. vinifera* resistant genotypes carrying the *Ren1* locus and the susceptible cv. ‘Carignan’ collected at 1 and 5 days after *E. necator* inoculation [75], and another comparing ‘Hunan-1’ (S) and ‘Shang-24’ (R) contrasting genotypes at 6, 24 and 96 hpi [76] (Suppl. Table 7). *VviNPR1* was found significantly up-modulated only at 6 hpi in ‘Shang-24’, together with *VviPR1*, whose up-modulation was maintained also at 24 and 96 hpi. *VviPR1* was significantly up-regulated also in ‘Carignan’ and three out of the seven *Ren1* genotypes, but at 5 dpi. *VviCOI1* and *VviMYC2* were significantly up-modulated only at 6 hpi in the susceptible ‘Hunan-1’. *VviWRKY51* was highly responsive in both Chinese genotypes at all time-points, and in ‘Carignan’ and three *Ren1* genotypes. *VviPR10-s11* was up-regulated in both Chinese genotypes at almost all time-points, and similarly *VviPR10-s10* or *VviPR10-s15* were modulated in ‘Carignan’ and all the Asian genotypes, in at least one time-point.

### Characterization of the grapevine Lipoxygenase gene family

The phylogenetic characterization of the LOX family was performed to improve the available description based on the advances in the versions of the grapevine reference genome and its annotation. In parallel, *LOXs* gene expression was evaluated in NY_39 and ‘Teroldego’ genotypes to investigate whether some members were modulated upon *E. necator* inoculation. A genome-wide search for *LOX* genes in the 12X.v2 genome assembly identified 13 non-redundant paralogues genes, confirming 12 paralogues already described [39] and finding a new one (*Vitvi07g01151*, see Suppl. Table 8). Nine are *13-LOXs* and include six highly similar (77.02%) members located in tandem on a 107.54 kb long genomic region on chromosome 6 (*Vitvi06g00149*, *Vitvi06g00150*, *Vitvi06g00153*, *Vitvi06g00155*, *Vitvi06g00156* on the + strand and *Vitvi06g00158* on the - strand). The four *9-LOXs* are located on chromosome 5 (*Vitvi05g00472*), chromosome 7 (*Vitvi07g01151*) and on chromosome 14 as two tandemly repeated paralogs (*Vitvi14g00234* and *Vitvi14g02539*) with 97.32% sequence similarity and only separated by 3.47 kb intergenic region. In *Arabidopsis*, six genes were identified, four 13-*LOXs* and two 9-*LOXs*, confirming previous results [77]. While in *Arabidopsis* all the 13-*LOXs* are chloroplastic [78], in grapevine there are also some members not containing the canonical plastidial transit peptide, according to in silico prediction. The presence of a signal peptide has been experimentally confirmed only for LoxA and LoxO [39, 79]. Manual curation of the gene models was required for some of them (Suppl. Table 8) and improved the phylogenetic analysis.

**Fig. 7:**
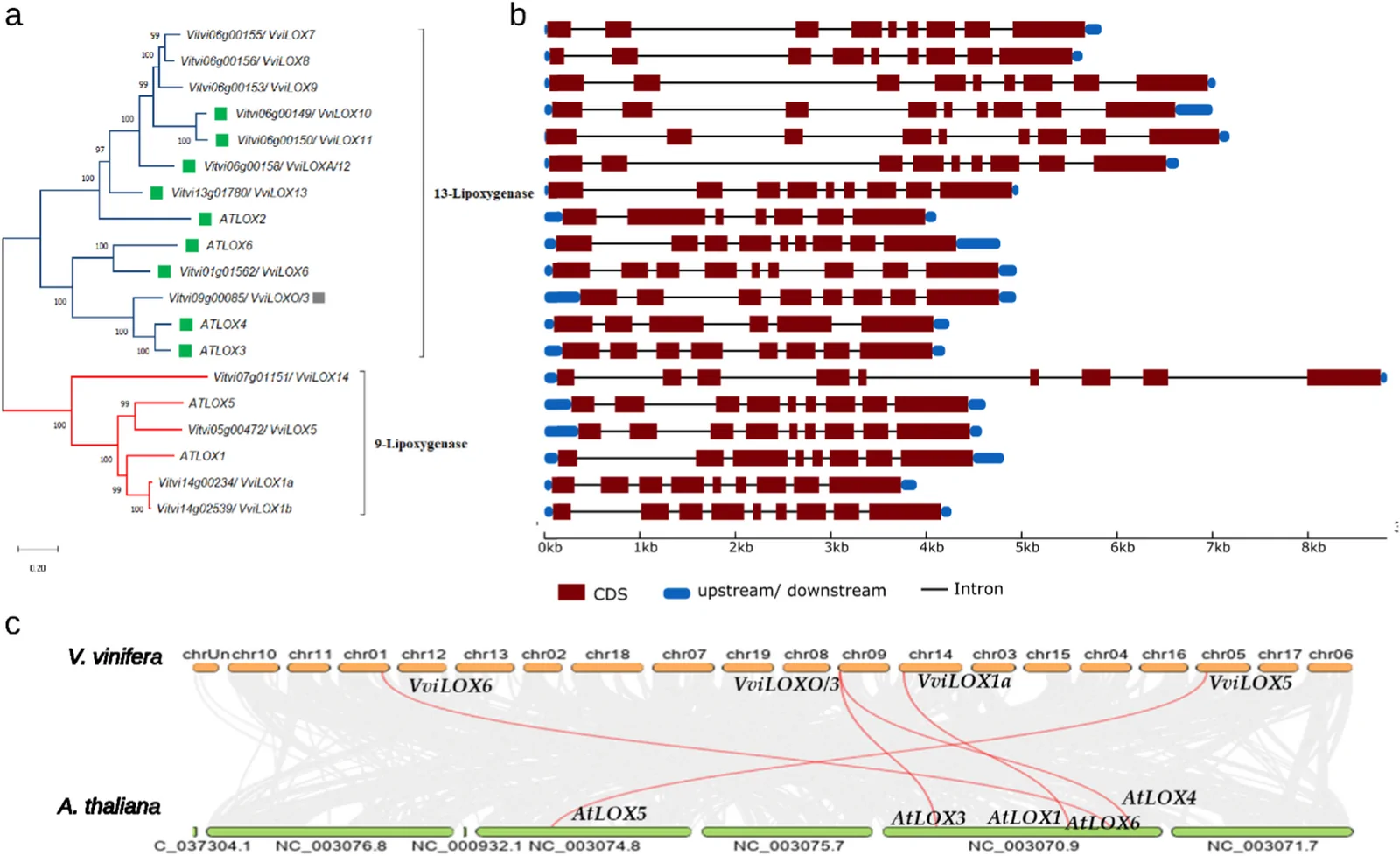
Phylogenetic tree and synteny analysis of the *V. vinifera *and *A. thaliana *lipoxygenase gene family. **a** Phylogenetic tree of *V. vinifera* and *A. thaliana* from LOX amino acid sequences multiple alignment. Evolutionary history was inferred using the maximum likelihood approach and by taking the tree with the highest likelihood. The green square indicates the presence of a predicted chloroplastic transit peptide (confirmed for *VviLoxA*), the grey square indicates the presence of a signal peptide (Podolyan et al., 2010). **b** Exon structure of grapevine and *Arabidopsis LOX* genes. **c** Synteny analysis

Multiple sequence alignment was used to determine similarity and generate a maximum likelihood phylogenetic tree to infer orthology between grapevine and *Arabidopsis LOXs* (Fig. 7a). The tree with the highest log likelihood (-15876.11) was selected and a robust branch support (bootstrap support ≥ 97%) was achieved for the associated taxa. The phylogenetic tree topology inferred the evolutionary separation of the 9- and 13-*LOXs* subfamilies with 52.01% average similarity between the two groups (Suppl. Table 9). Furthermore, the 13-*LOXs* were separated into two evolutionary lineages: one clade containing three *Arabidopsis* (*AtLOX3*,*4* and *6*) and *Vitvi09g00085* and *Vitvi01g01562* and another clade formed by *AtLOX2*, *Vitvi13g01780* and the grapevine-specific chromosome 6 tandem duplications. The latter group constituted several isoform pairs forming paralogs with extremely high similarity index, such as *Vitvi06g00149* and *Vitvi06g00150* (> 90%) and *Vitvi06g00155* and *Vitvi06g00156* (91.14%). On the other hand, the 9-*LOXs* fell into a single clade formed by two *Arabidopsis* (*AtLOX1* and *5*) and four grapevine *LOXs* (*Vitvi07g01151*, *Vitvi05g00472*, *Vitvi14g00234* and *Vitvi14g02539*). The overall average similarity of the group was 72.04% including *Vitvi07g01151*, which showed the lowest average similarity (59.29%) with the other members of the group. The exon structure of grapevine *LOXs* was highly conserved within all the genes with nine exons, except *VviLOXO* (Fig. 7b). On the other hand, the number of exons in *Arabidopsis* genes ranged from six to nine. Remarkably, synteny analysis highlighted the absence of orthologous genes as well as synchronous chromosomal region for the cluster on chromosome 6 (Fig. 7c).

**Fig. 8:**
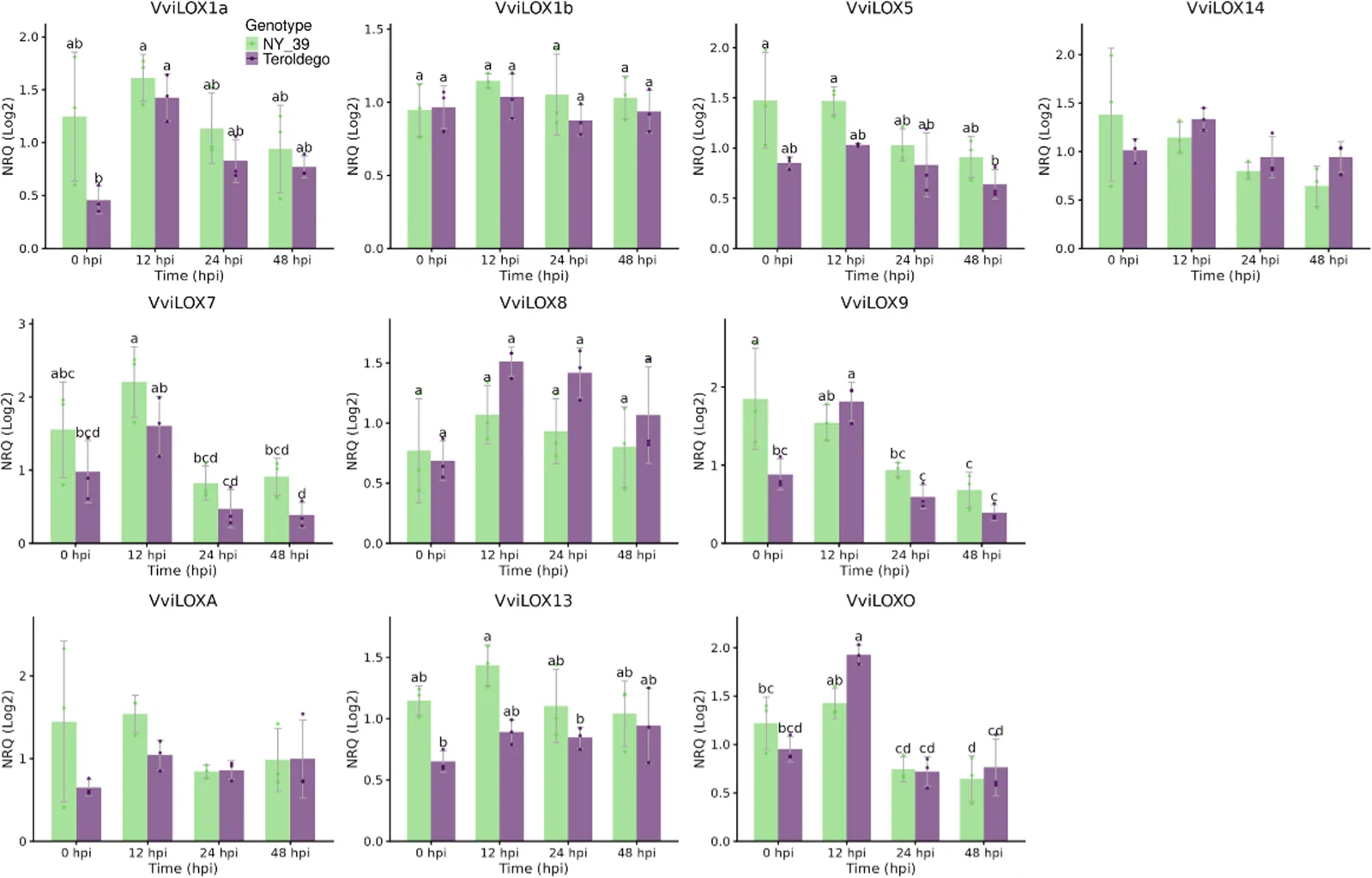
qPCR analysis of grapevine LOX genes upon *E. necator* inoculation. Dots represent biological replicates (*n* = 3), while bars and whiskers respectively the mean value and the standard deviation (SD). Statistical significance was assessed using a two-way ANOVA with Tukey multiple comparisons test on log_2_ transformed normalized relative quantity (NRQ), p-value < 0.05, only for the genes passing the Shapiro-Wilk normality test

Due to the high homology shared by the *LOX* members, before performing gene expression analysis a preliminary assay was carried out to select primers with single and sharp melting peaks (Suppl. Figure 1). *LOXs’* expression analysis in the resistant and the susceptible genotypes during *E. necator* infection showed that in the clade of 9-*LOXs*, only *VviLOX1a* showed a significant induction at 12 hpi in ‘Teroldego’, while no change was detected for *VviLOX1b* - despite their high similarity (97.32%)- *VviLOX5*, and *VviLOX14* (Fig. 8). Regarding the cytosolic 13-*LOXs*, of the three paralogs of the tandem repeat on chromosome 6, only *VviLOX7* and *VviLOX9* were modulated (Fig. 8). *VviLOX7* was significantly down-regulated between 12 and 24 hpi in both genotypes; *VviLOX9* basal expression was higher in NY_39 and decreased at 24 hpi while in ‘Teroldego’ the gene was induced at 12 hpi and then down-regulated at 24 hpi. The chloroplastic *VviLOXO* showed a modulation similar to *VviLOX9*, although its basal expression was not significantly different in the two genotypes (Fig. 8).

## Discussion

The urgency to develop valuable germplasm for a more sustainable viticulture encouraged crossbreeding programs to apply *R*-loci pyramiding for a more durable resistance to fungal pathogens. However, this new material needs to be characterized, as the genetic backgrounds to which the *R*-loci are transferred can affect the defence response as well [75]. In the current study, the PM response in a complex hybrid parental line carrying *Run1*, *Ren2*, *Ren3*, and *Ren9* loci - derived by the American *M. rotundifolia*, *V. cinerea*, and *V. rupestris* respectively - was compared with a susceptible landrace. Microscopic imaging indicated the occurrence of an early and fast post-penetration response in NY_39, as hyphal growth was completely suppressed after primary appressoria formation (at 24 hpi), thus determining complete resistance as assessed at 15 dpi. These observations are consistent with a study comparing genotypes carrying the single *Run1* and *Ren1* or a combination of both, where rapid PCD, ROS accumulation and callose deposition at the fungal penetration site occurred more rapidly in the two genotypes carrying the *Run1* locus [80]. Our study dissects and supports the prominent effect of the *Run1* locus over other *Ren* loci.

### Lipid dynamics and peroxidation as early markers of host resistance to powdery mildew in grapevine

As an obligate biotrophic pathogens, *E. necator* relies exclusively on its host for carbon and nitrogen supply; consequently, it actively modulates plant metabolic processes to satisfy its nutritional requirements [40, 81]. The fungus grows epiphytically on the upper leaf surface and develops specialized feeding structures, termed haustoria, inside the host epidermal cells, where effectors, enzymes, and small molecules necessary for the pathogen growth and reproduction are exchanged [2]. *E. necator* lost key genes for fatty acids and sterol biosynthesis, thus making lipids a critical factor in the host-microbe interaction [76]. A primary distinction between the resistant hybrid NY_39 and the susceptible cultivar ‘Teroldego’ concerned the TG content at 12 hpi, which significantly decreased in the former and increased in the latter. This divergence in storage lipid dynamics may represent a marker of the early metabolic reprogramming that correlates with host resistance. At the transcriptomic level, previous studies have reported the down-regulation of enzymes involved in *de novo* fatty acid biosynthesis and elongation as early as 4–6 hpi in resistant genotypes, coupled with the up-regulation of enzymes dedicated to lipid degradation [71, 76]. These transcriptional shifts indicate a mobilization of fatty acid carbon reserves back to the key precursor, acetyl-CoA, which correlates with the up-regulation of the isoprenoid pathway during PM infection [71]. TG degradation could compensate for the reported decline in photosynthesis and supply to plant metabolic demand. Conversely, in the susceptible hosts, it seemed that *E. necator* succeeded in modulating plant lipid metabolism for its own benefit. A study characterizing the interaction between *Medicago truncatula* and arbuscular mycorrhizal fungi describes a pathway of fatty acyl groups export from the plant to the fungi highlighting the nutritive function of lipid exchange [82]. A second divergence between the genotypes was the tendential decrease in MGDG galactolipids observed in NY_39 at 12 hpi (and at 24 hpi in ‘Teroldego’), correlating with a marked increase in chloroplastic membrane peroxidation at the same time-point in NY_39, which can function as a signalling mechanism [83, 84]. We previously reported a similar behaviour in resistant maize lines inoculated with *Fusarium verticillioides* [23], reinforcing the hypothesis that the activation and timing of the defence signalling are decisive factors in the outcome of the plant-pathogen interaction. Finally, the accumulation of phosphatidic acid (PA) at 48 hpi in ‘Teroldego’ occurred when the infection was well-established, potentially representing late-stage signalling for the activation of alternative defence-related enzymes [85]. In contrast, the absence of PA accumulation in NY_39 at 48 hpi suggests that the main defence response has been successful and basal cell metabolism has been restored.

### Hormonal and molecular signatures of the defence response

Statistically supported changes were observed for SA, SA-glucoside, *cis*-OPDA, OH-JA and ABA. The involvement of ABA in plant defence, still controversial, has not been further investigated in this study. SA typically accumulates at the infection site following the recognition of pathogen effector by specific receptors, mediating the defence response to biotrophic pathogens [86]. In this study, the resistant genotype showed a higher basal level of SA and of its storage form SA-glucoside. Although a very small increase at 12 hpi was observed in both genotypes, it was not statistically supported; thus we can only speculate that our “whole leaf” or “bulk” experimental approach might have prevented us from detecting rapid SA gradients confined to the immediate area surrounding the appressorium penetration site [15]. Higher basal levels in resistant genotypes have also been observed also for *V. aestivalis* and the PM tolerant *Vitis* hybrid cv. ‘101 − 14 Millardet et de Grasset’ [71, 87]. We did not observe modulation of the SA receptor *VviNPR1* and its target *VviPR1*. While both genes were found up-regulated very early (6 hpi) in the resistant ‘Shang-24’ genotype [76], *VviPR1* was found up-regulated also at 24 and 96 hpi in ‘Shang-24’ and very late (5 dpi) in the susceptible ‘Carignan’ and in 3 out of the 7 Asian resistant genotypes [75], showing a complex pattern of modulation.

Regarding jasmonate signalling, levels of *cis*-OPDA (a precursor of JA synthesis) significantly decreased in NY_39 upon pathogen inoculation. Conversely, the hydroxylated OH-JA, not modulated by the pathogen in either genotypes, was measured at higher levels in ‘Teroldego’ than in NY_39 at both basal conditions and 24 hpi. These results align with Amaro et al. (2023), who also reported minimal modulation of JA-related compounds [87]. In contrast, significant increase in JA-Ile at 12 hpi is reported for the resistant ‘Regent’ in response to *P. viticola*, supporting the role of JA in biotrophic oomycete defence [31]. In *Arabidopsis*, during effector-triggered immunity following plant-pathogen interaction, JA synthesis and signalling pathways have been shown to be triggered independently of the canonical route involving the *COI1* JA-Ile receptor. In fact, an alternative mechanism has been observed, involving the SA receptors *NPR3/NPR4* which promote the degradation of JAZs, repressors of the JA signalling [88]. This alternative mechanism, besides the less abundant and unchanged content of JA-Ile, could explain the lack of *VviCOI1* modulation observed in our study, as well as in the *V. vinifera* Asian resistant accessions and in the susceptible cv. ‘Carignan’ [75]. Actually, the transient activation of the *VviCOI1-MYC2* pathway at 6 hpi correlates with susceptibility in the contrasting Chinese genotypes [76].

**Fig. 9:**
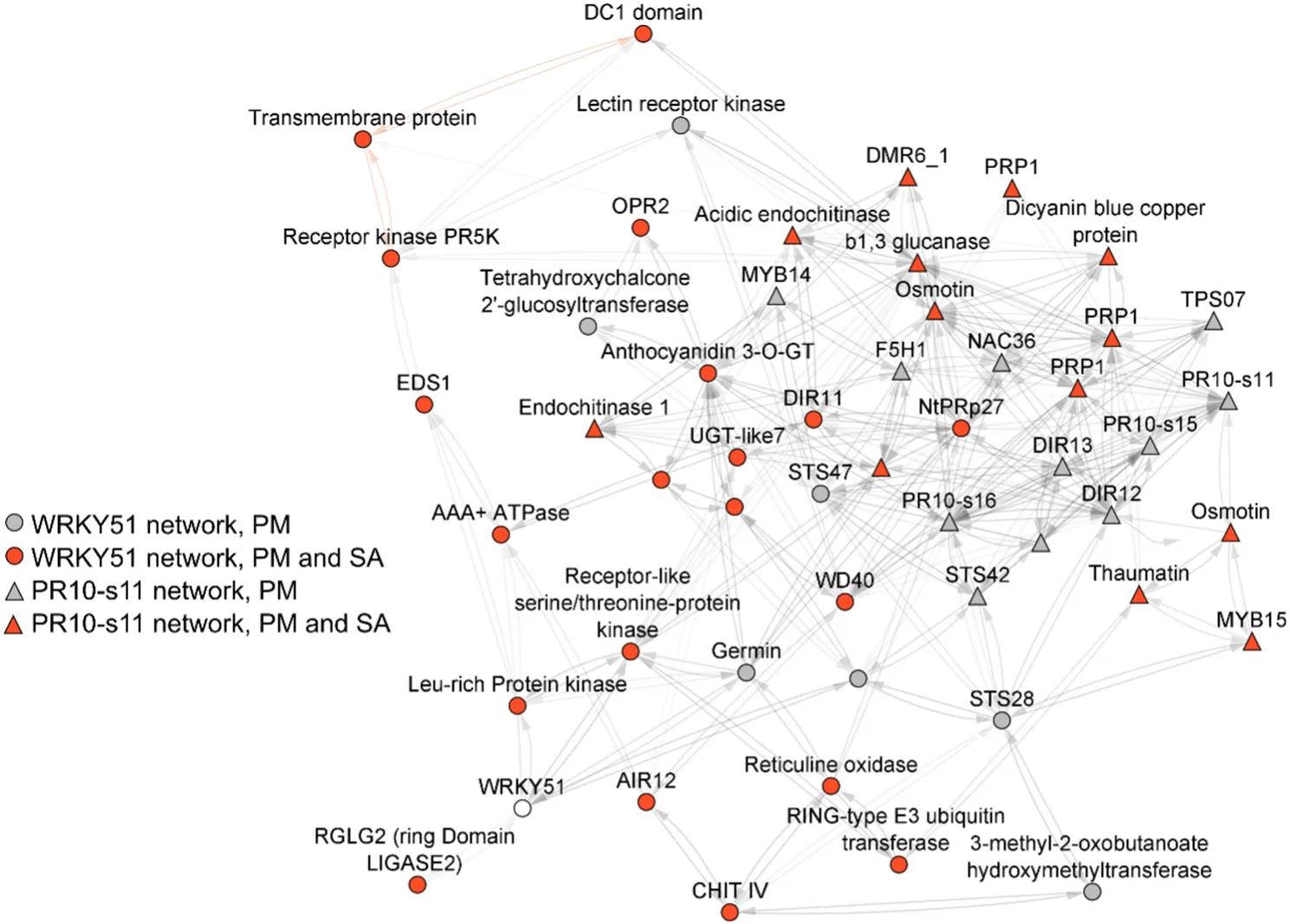
Gene network analysis of *VviWRKY51* and *VviPR10-s11*. Triangle and circle node shapes indicate *VviPR10-s11* and *VviWRKY51* OneGenE networks, respectively. Red and grey colours correspond to SA-dependent and independent PM-responsive genes, respectively. Unlabelled nodes refer to unknown genes, *VviWRKY51*was not present on the microarray used in [40], so there is no information about its PM or SA-dependence from this study

Beyond the complex SA-JA interplay occurring upon fungal infection at the infection site [15], Toth et al. (2016) identified a set of PM-responsive genes in the susceptible cv. ‘Cabernet Sauvignon’ that do not require SA: these include many *PR10* isoforms, coding for nucleases critical in mediating fungal resistance [73], stilbene synthases, Dirigent proteins, *VviMYB14* and ferulate-5-hydroxylase, involved in oligomeric stilbenoid and lignin synthesis [40]. Given the high modulation of *VviPR10-s11* and *VviWRKY51* in both NY_39 and ‘Teroldego’, a gene network analysis with OneGenE [63] was performed to gain more insight into their function based on their correlated genes, and the SA-dependence information from [40] was integrated into the network visualization (Fig. 9).

The OneGenE network confirmed a tight association between the SA-independent modulation of PR10 nucleases and enzymes involved in phytoalexins production (grey triangles). *VviWRKY51* was found more connected to receptors related to SA signalling and thus possibly involved in pathogen perception and response. While *VviPR10-s11* and *VviWRKY51* gene expression profiles followed similar trends in both genotypes- in line with many transcriptomic studies comparing susceptible and resistant genotypes [71, 89]- metabolic studies have identified specific stilbenoids accumulation, such as pallidol and astringin, exclusively in resistant genotypes like BC4 (carrying *Run1*) and ‘Bianca’ (carrying *Ren3* and *Ren9*) [32]. Other stilbenoids, such as pterostilbene and viniferin, correlate with the presence of *Rpv3-1* (‘Regent’, ‘Bianca’), *Rpv3-3* (‘Merzling’) and *Rpv10* (‘Solaris’) resistance loci for *P. viticola* [90–92] supporting a broad-spectrum anti-fungal resistance mechanism based on phytoalexins. As a multi-resistant genotype carrying also the *Rpv3-1* locus, NY_39 is likely to accumulate stilbenoids with antifungal activity.

Finally, the constitutive higher expression of the *VviAMP1* defensin in NY_39 represents a significant novelty, as its role during *E. necator* infection had not been previously investigated. Defensins are small, cysteine-rich cationic proteins and integral components of the plant innate immune system that exhibit potent antimicrobial activity against a broad spectrum of pathogens [93]. They possess a wide array of biological activities, including antimicrobial and insecticidal properties, as well as protein synthesis inhibition capabilities; their introgression into transgenic plants has yielded encouraging results [94]. In grapevine, further studies are needed to evaluate their potential as bio-fungicides, aligning their use with the goal of a more sustainable agriculture.

### The Lipoxygenase family and their modulation in response to PM

Lipoxygenases-mediated lipid peroxidation plays an important role in pathogen perception and response, as it represents the first step in either the synthesis of the JA and oxylipin signal mediators [18] or compounds with antimicrobial activity [95]. In maize their involvement in defence has been widely reported [23, 96, 97]. Here, we updated the annotation of the gene family, identifying a new gene on chromosome 7 and manually curated the gene models, thus improving the phylogenetic analysis and gene annotation. A cluster of highly homologous *LOXs* located on chromosome 6 does not have an ortholog in *Arabidopsis.* Except for *VviLOXA*, which is located on the antisense strand and codes for a chloroplastic protein [34], the other genes do not present a target peptide, likely coding for cytosolic proteins. *VviLOXA* preferentially peroxidizes galactolipids rather than free fatty acids [98] and could thus be responsible for the observed basal galactolipid peroxidation. Conversely, the chloroplastic *VviLOXO* is significantly up-modulated at 12 hpi in ‘Teroldego’ and down-modulated at 24 and 48 hpi in both genotypes. This gene, previously found up-regulated in berries inoculated with *B. cinerea* [39], is a candidate for JA biosynthesis. Although we didn’t measure an increase in JA, we cannot exclude its rapid conversion into methyl-JA. Concerning the cluster of cytosolic *LOXs*, *VviLOX7* and *VviLOX8* were found up-regulated also in pre-véraison berries infected with PM [45], and in the resistant ‘Shang-24’ genotype (Suppl. Table 7b). We observe the induction of *VviLOX9* at 12 hpi in ‘Teroldego’ and a higher basal level in NY_39, and a down-regulation of *VviLOX7* at 24 and 48 hpi in both genotypes. Together with the transient induction of the cytosolic 9-LOX *VviLOX1a* at 12 hpi in ‘Teroldego’ they may trigger the production of 9- and 13-HODE and HOTrE derived oxylipins and volatiles either involved in plant-pathogen interaction or in plant signalling.

## Conclusions

The present study highlights constitutive differences and divergent pathogen-induced molecular events occurring during the *E. necator*–grapevine interaction in a multi-loci resistant genotype versus a susceptible cultivar. The rapid, post-penetration arrest of pathogen invasion in the resistant genotype correlates with the presence of effector recognition receptors and a higher basal SA content. Furthermore, resistance is associated with the constitutive expression of the defensin *VviAMP1*, rapid galactolipid peroxidation and the induction of a broad defence response, involving *PR10* nucleases, usually co-expressed with enzymes of the stilbenoid and lignin metabolism. Distinct shifts in lipid metabolism suggest that while the pathogen may manipulate storage lipids during compatible interactions, the resistant host may utilize them as a source of acetyl-CoA for defence. Although candidate *LOX* genes for chloroplastic lipid peroxidation and cytosolic oxylipin synthesis were proposed, their specific roles warrant further investigation. A deeper understanding of these interactions will facilitate the development of durable, host-mediated resistance in grapevine crossbreeding programs.

## Supplementary Information

Supplementary Material 1. Supplementary Table 1: List of the Internal standards (IS) used for the semi-quantitative targeted lipidomic analysis. Supplementary Table 2: Identification of oxidized lipids. Supplementary Table 3:  Lipid species measured within each lipid class. Supplementary Table 4: Primer sequences. Supplementary Table 5:  Lipidomic data. Supplementary Table 6: Lipid modulation after E. necator inoculation. Supplementary Table 7: Gene expression modulation from RNA-seq analyses performed in the studies Amrine et al. (2015) and Jiao et al. (2021).  Supplementary Table 8: The LOX gene family. Supplementary Table 9: Similarity matrix among grapevine LOX isoforms.

Supplementary Material 2. Supplementary Figure 1: Checking the specificity of the primer pairs to be used for analysing VviLOX genes expression. Supplementary Figure 2: Overview of the lipid composition of leaf samples taken from ‘Teroldego’ and NY_39 plants before E. necator inoculation. Supplementary Figure 3:  Profiles of the more abundant molecular species within the most modulated classes, in NY_39 and ‘Teroldego’ upon E. necator inoculation. Supplementary Figure 4: Overview of galactolipid peroxidation in leaf samples taken from ‘Teroldego’ and NY_39 plants before E. necator inoculation.

## Data Availability

The datasets generated during the current study are available as Additional Electronic Material.

## Abbreviations

CAR: Carnitine
DG: Diacylglycerol
DGDG: Digalactosyldiacylglycerol
dhCER: Dihydroceramide
dpi: Days post-infection
FFA: Free fatty acid
hpi: Hours post-infection
LPA: Lysophosphatidic acid
LPC: Lysophosphatidylcholine
LPE: Lysophosphatidylethanolamine
LPI: Lysophosphatidylinositol
LPG: Lysophosphatidylglycerol
MGDG: Monogalactosyldiacylglycerol
OIV: Organisation Internationale de la Vigne et du Vin
13-HOTrE: 13(S)-hydroxyoctadeca-9Z,11E,15Z-trienoic acid
PA: Phosphatidic acid
PC: Phosphatidylcholine
PE: Phosphatidylethanolamine
PI: Phosphatidylinositol
PG: Phosphatidylglycerol
PS: Phosphatidylserine
REN: Resistance to Erysiphe necator
RUN: Resistance to Uncinula necator
SP: Sphingolipid
TG: Triacylglycerol
