# Supplementary material for: Distinct lipid and hormonal signatures reveal divergent responses to Erysiphe necator in resistant and susceptible grapevine genotypes
Source: BMC Plant Biol. 2026 Aug 3;26:1854. doi: 10.1186/s12870-026-09654-9 (PMC13721609; doi:10.1186/s12870-026-09654-9)
Supplement: Supplementary file 1 [file 12870_2026_9654_MOESM2_ESM.pdf]

Distinct lipid and hormonal signatures reveal divergent responses to *Erysiphe necator* in resistant and susceptible grapevine genotypes

Authors

Mikias D. Guche[1,2,3], Lorenza Dalla Costa[2], Alessandra Lanubile[3], Axel Mithöfer[4], Domenico Masuero[2], Francesco Trenti[2,5], Mar Garcia-Aloy[2], Silvia Vezzulli[2], Urska Vrhovsek[2], Graziano Guella[5], Mickael Malnoy[2], Adriano Marocco[3], Stefania Pilati[2§].

[1] C3A - Centro Agricoltura Alimenti Ambiente, University of Trento, Via Edmund Mach 1, 38098 San Michele all'Adige, Italy

[2] Research and Innovation Centre, Fondazione Edmund Mach, Via E. Mach 1, 38098 San Michele all'Adige, Italy

[3] Department of Sustainable Crop Production, Università Cattolica del Sacro Cuore, Via Emilia Parmense 84, 29122 Piacenza, Italy

[4] Research Group Plant Defense Physiology, Max-Planck-Institute for Chemical Ecology, Hans-Knöll-Str. 8 D-07745 Jena, Germany

[5] Department of Physics, University of Trento, Via Sommarive 14, 38123 Povo, Italy

[§] Corresponding author: stefania.pilati@fmach.it

		Amplicon lenght		Melting temperature of amplicon		Selected primer pair
**Graph**	**LOX ID**	**Short**	**Long**	**short**	**long**	
1	Lox 13	119	168	79.00	84.00	short
2	Lox O	116	157	78.00	79.00	short
3	Lox 8	108	166	79.50	No amplification	short
4	Lox 5	118	163	80.00	81.00	short
5	Lox A	114	161	83.00	No amplification	short
6	Lox 14	121	156	81.00	80.50	short
7	Lox 7	118	161	81.00	81.00	short
8	Lox 1b	108	166	78.50	No amplification	short
9	Lox 9	114	167	80.00	79.50	long
10	Lox 1a	103	170	76.50	81.00-79.50	short

Graph 1 Graph 2 Graph 3

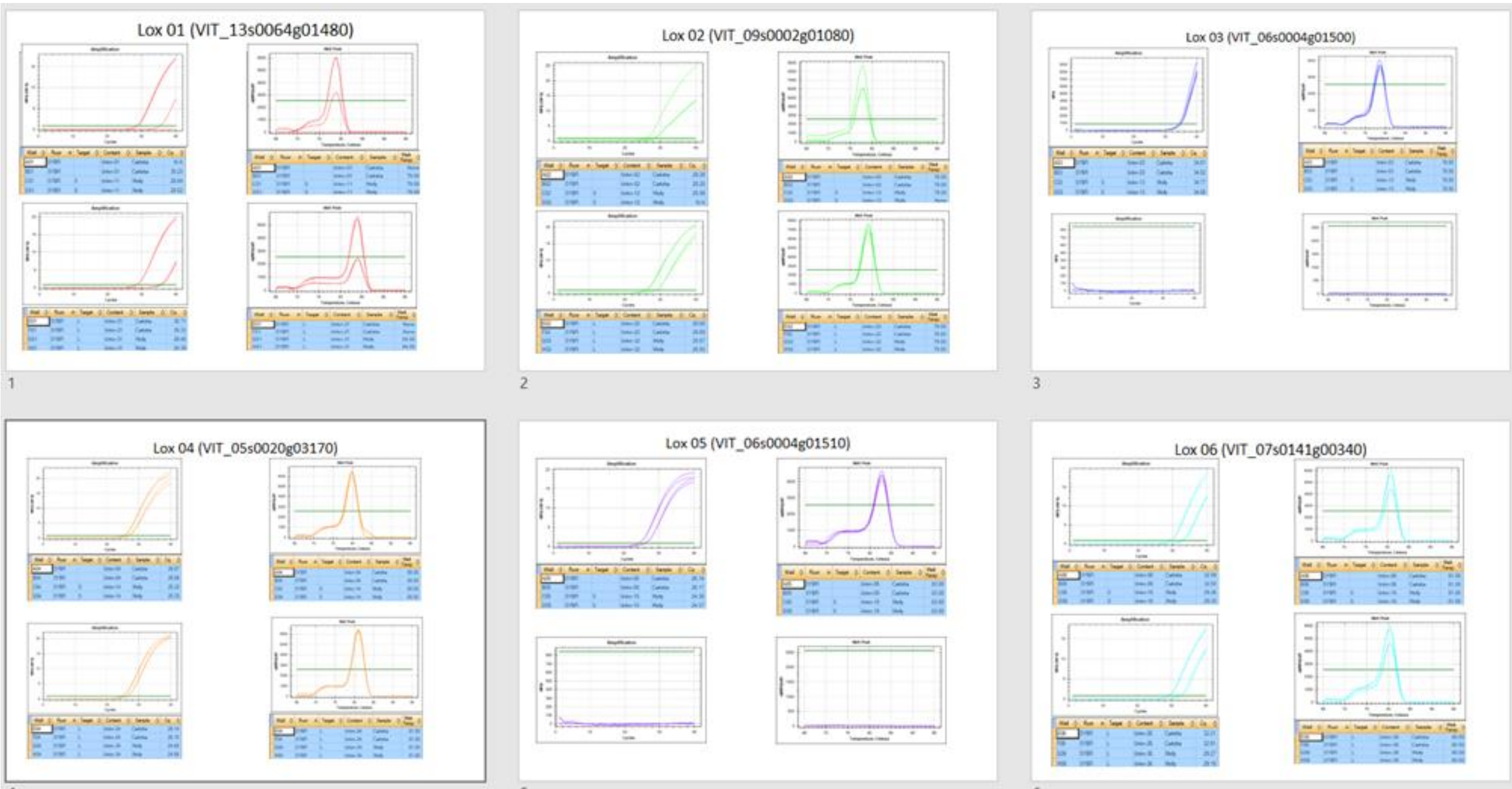

Graph 4 Graph 5 Graph 6

Graph 7 Graph 8 Graph 9

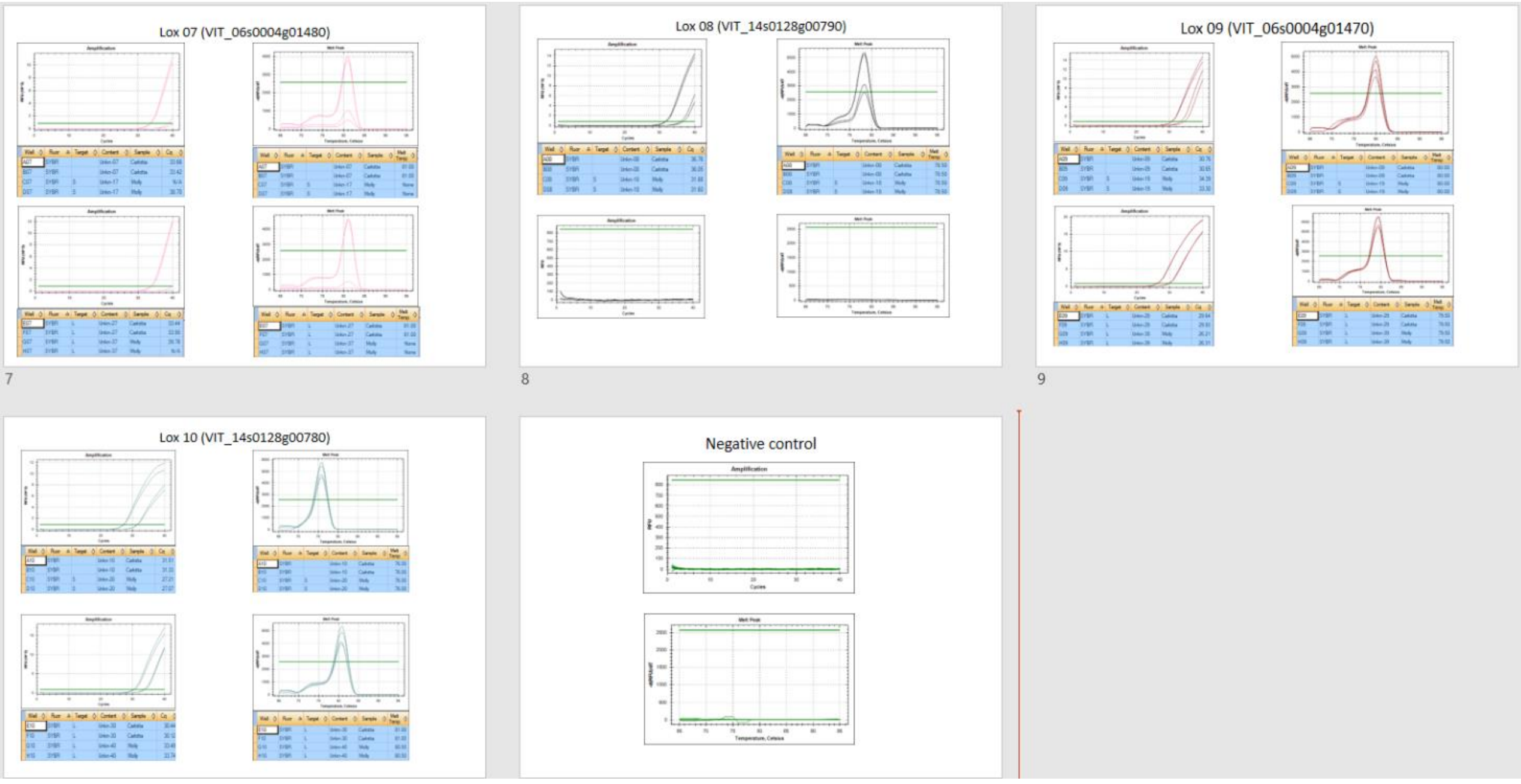

Graph 10

Supplementary Figure 1. Checking the specificity of the primer pairs to be used for *VviLOX* genes expression analysis. The table shows the lengths and melting temperatures of the amplicons produced using two primer pairs for each *LOX* gene. The final column indicates which amplicon, and therefore which primer pair, was selected for subsequent analysis. In the ten graphs associated with the *LOX* genes, the amplification curves are shown on the left and the melting curves on the right. Primers that amplify the short or long regions were used in the top and bottom panels, respectively. Two cDNA templates - obtained from leaves of cv. 'Sugraone' grown in the greenhouse and in the field - were used, in two technical replicates.

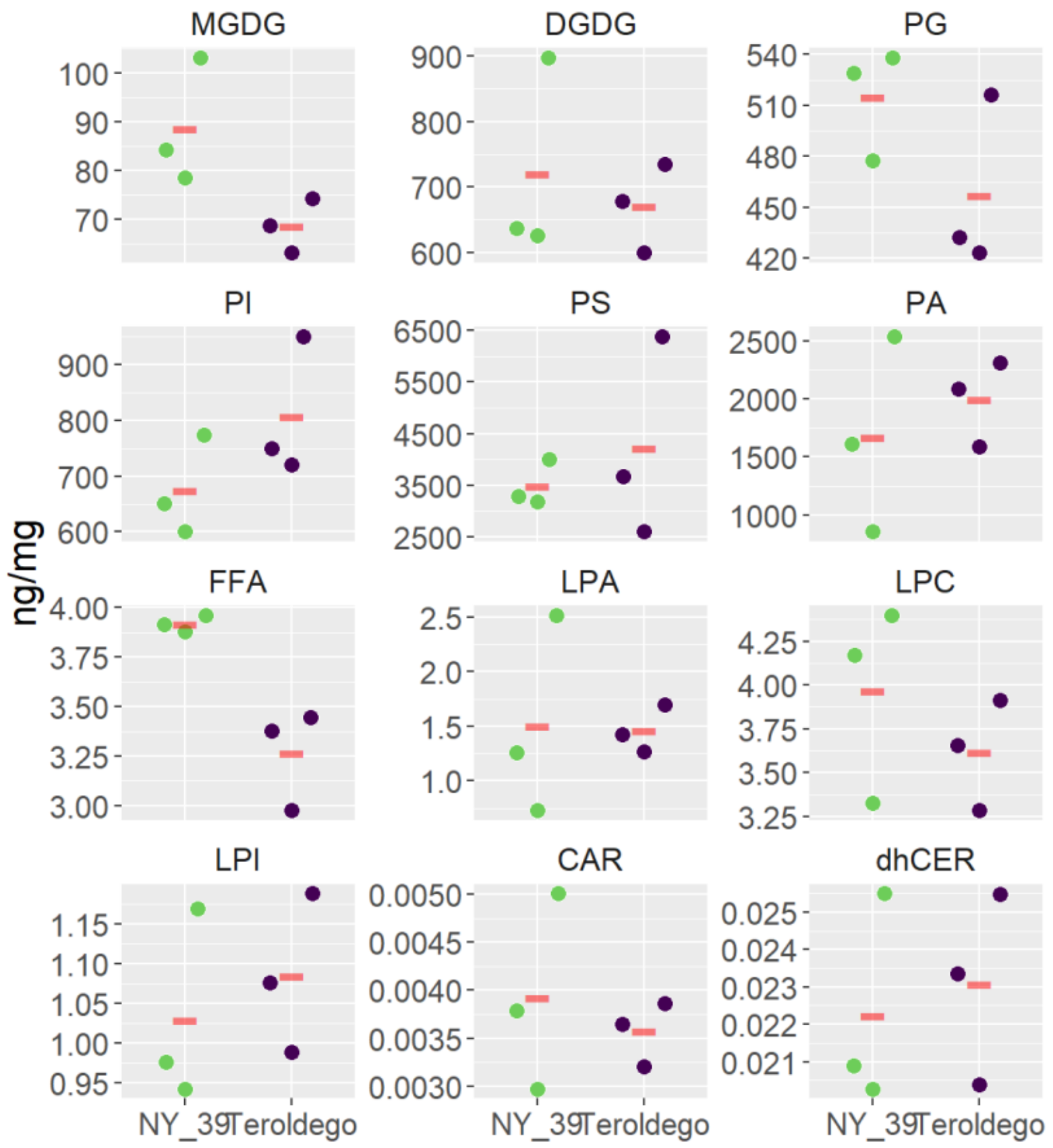

Supplementary Figure 2: Overview of the lipid composition of leaf samples taken from NY_39 and 'Teroldego' plants before *E. necator* inoculation. The sum of the amount of each species within a class is reported as dots for each biological replicate for each genotype (green dots for NY_39, purple dots for 'Teroldego'). Amount is expressed in ng/mg FW, red horizontal lines indicate mean values.

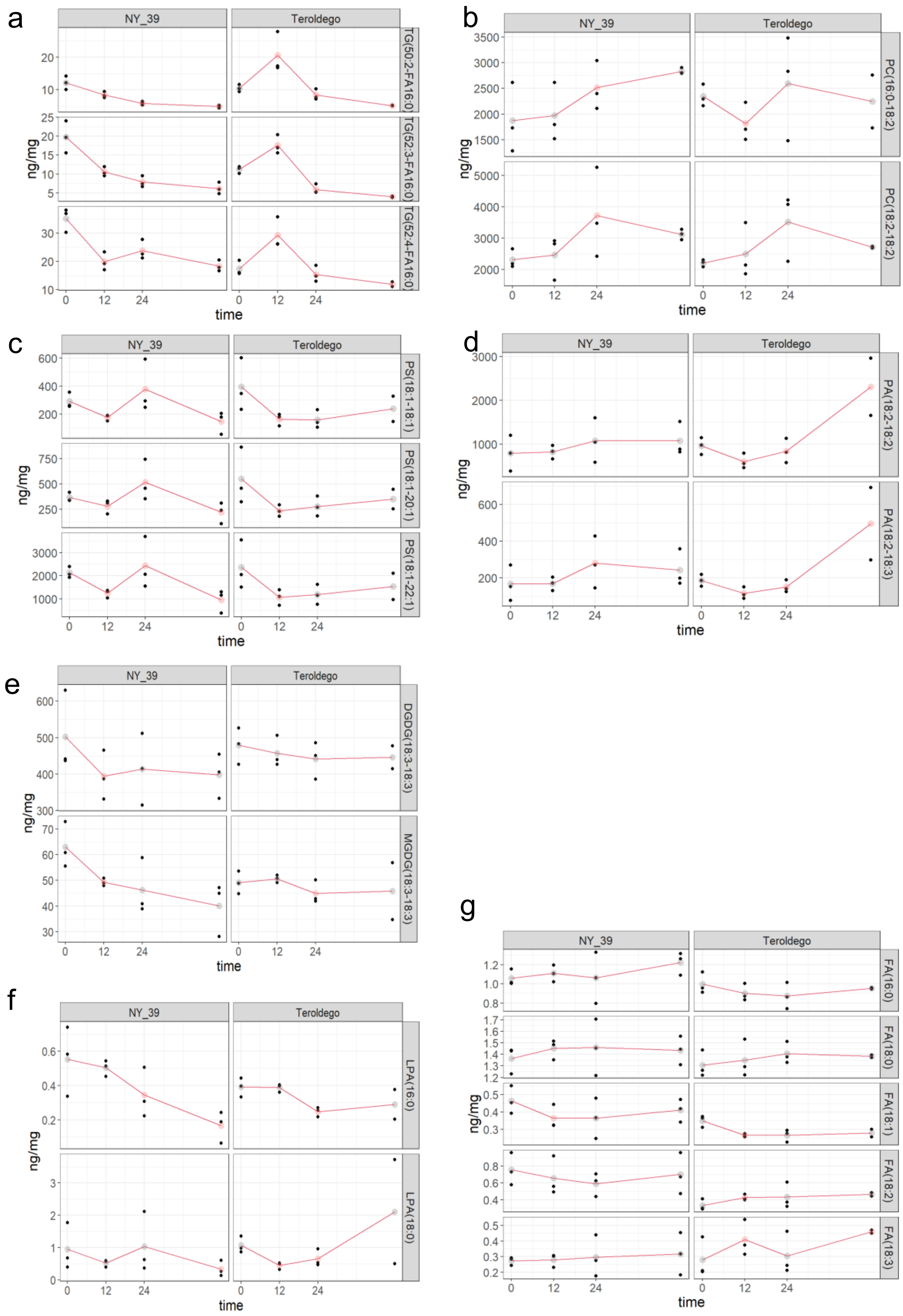

Supplementary Figure 3: Profiles of the more abundant molecular species within the most modulated classes upon *E. necator* inoculation, in NY_39 and 'Teroldego'. Lines connect mean values of each time point; mean values are indicated as red dots when the abs (ES) of the comparison of that time point versus the previous one is >1, as grey dots otherwise.

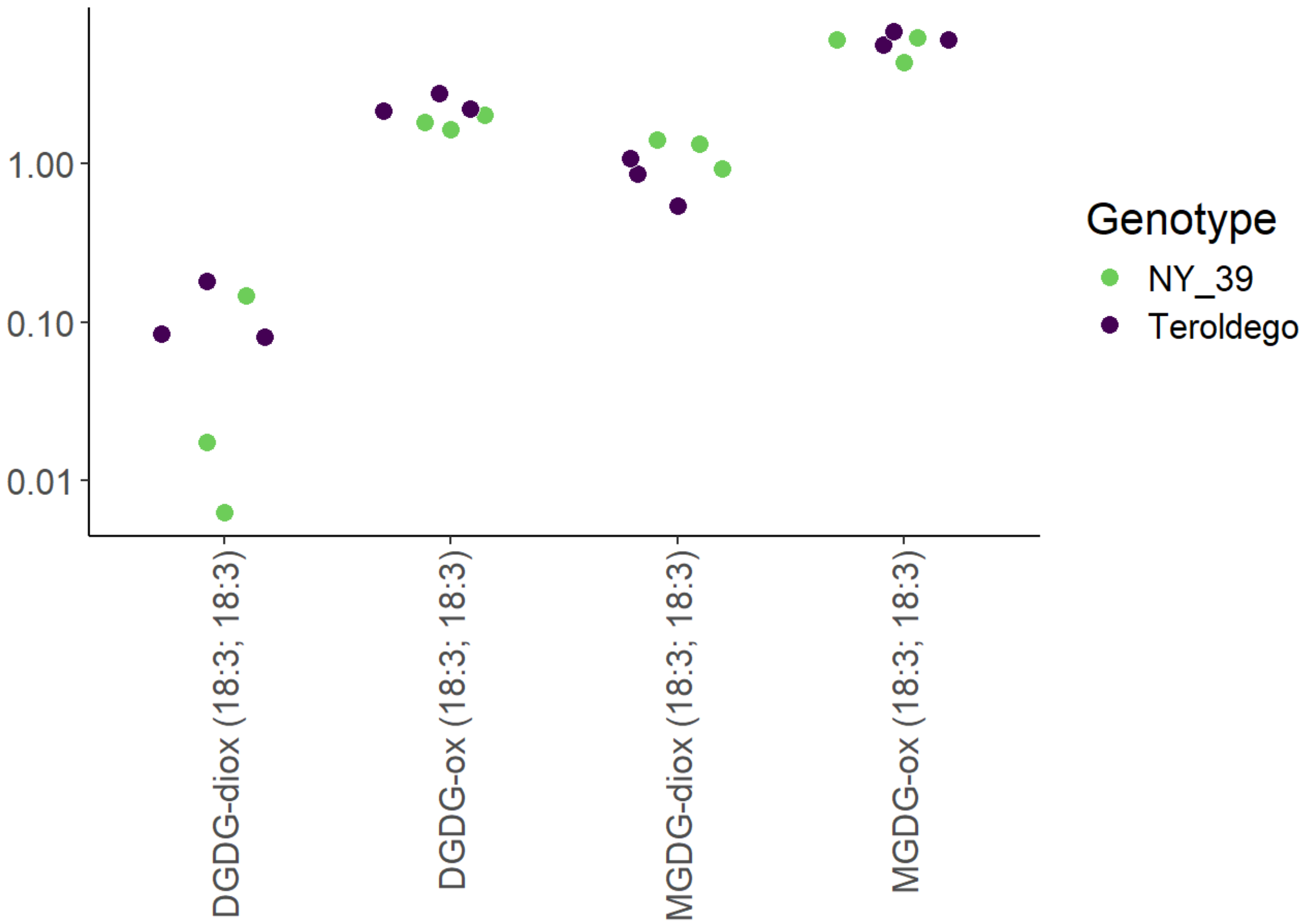

Supplementary Figure 4: Overview of galactolipid peroxidation in leaf samples taken from NY_39 and 'Teroldego' plants before *E. necator* inoculation. The peroxidized species are expressed as relative amount (%) within each species, i.e. DGDG 36:6 and MGDG 36:6.
